# Carbonic anhydrases contribute to mitochondrial function, conidial development, and pathogenicity of *Magnaporthe oryzae*

**DOI:** 10.1128/aem.02488-25

**Published:** 2026-02-12

**Authors:** Qingqing Cui, Tingzhen Wang, Yujia Li, Xiaojing Liang, Xiaotong Song, Xinyue Ji, Linyi Wang, Xinquan Wang, Zhentao Chu, Yuejia Dang, Shi-hong Zhang

**Affiliations:** 1 College of Life and Health, Institute of Modern Agriculture Research in Dalian University, Dalian, China; 2 College of Plant Protection, Shenyang Agricultural University 98428, Shenyang, China; The University of Arizona, Tucson, Arizona, USA

**Keywords:** *Magnaporthe oryzae*, carbonic anhydrases, mitochondria, nitrogen metabolism

## Abstract

Carbonic anhydrases (CAs) are metalloenzymes widely present in biological systems, capable of catalyzing the reversible hydration of carbon dioxide (CO₂) to form bicarbonate (HCO_3_⁻) and protons (H^+^). This biochemical reaction plays a crucial role in various physiological processes. Previous studies have shown that MoCA1, a carbonic anhydrase in *Magnaporthe oryzae*, is localized in the mitochondria and is involved in conidial development and pathogenicity. However, the functions of other carbonic anhydrases in *M. oryzae*, such as MoCA2, MoCA4, and MoCA6, remain largely unexplored. Through subcellular localization analysis using DsRED-tagged fusion proteins, it was found that MoCAs are also localized in the mitochondria, and they exhibit interactions within this organelle. When the genes encoding *MoCAs* were deleted, the resulting mutants Δ*MoCA2*, Δ*MoCA4*, and Δ*MoCA6* exhibited significant defects in conidial development and pathogenicity. Additionally, these mutants showed a marked decrease in mitochondrial membrane potential, indicating an apoptotic state, along with reduced intracellular ATP levels and downregulated expression of genes related to ATP synthesis. In nitrogen metabolism studies, the mutants displayed corresponding phenotypic changes, with significant downregulation of nitrogen metabolism-related gene expression. Taken together, the results of this study demonstrate that mitochondrial MoCAs play a critical role in conidiation and pathogenic mechanisms in *M. oryzae*. They function by coordinating the regulation of mitochondrial function, ATP synthesis, and the glutamine-glutamate metabolic pathway, thereby maintaining cellular pH homeostasis. These results provide important theoretical insights for the identification and development of novel targets for the control of *M. oryzae*.

**IMPORTANCE:**

Rice blast disease, caused by the fungal pathogen *Magnaporthe oryzae*, represents a major threat to global rice production, resulting in significant yield losses annually. During infection, *M. oryzae* encounters multiple environmental stresses within the host, such as nitrogen limitation, elevated bicarbonate (HCO_3_⁻) levels, and hypoxic conditions. These challenges necessitate robust mechanisms to maintain intracellular pH and metabolic stability, which are crucial for pathogen survival and virulence. Our study highlights the essential role of the carbonic anhydrase family of enzymes in regulating intracellular pH homeostasis in *M. oryzae*. These findings provide valuable insights into the molecular strategies employed by plant pathogens to adapt to hostile host environments, potentially informing the development of novel disease management approaches.

## INTRODUCTION

Carbonic anhydrases (CAs) are metal-containing enzymes and CO₂-binding proteins, which catalyze the reversible hydration reaction of carbon dioxide (CO_2_) (1, 2). CAs are ubiquitous in three types of organisms: archaea, bacteria, and eukarya, where the enzymes play a crucial role in a number of essential vital processes, such as maintaining acid-base homeostasis, regulating fluid balance and cellular pH, participating in CO_2_ transport, carbon fixation, respiration and photosynthesis, and cell metabolism (3–9). CAs are divided into eight classes (α, β, γ, δ, ζ, η, θ, and ι), among which α-CAs are the largest class and exist in animals, bacteria, protozoa, fungi, and photosynthetic organisms (10). In protozoa, fungi, and bacteria, α-CAs supply the bicarbonate (HCO_3_⁻) required for metabolism and participate in pH regulation (3). β-CAs have been identified in the mitochondria of algae and higher plants (11), where they are critical for plant growth and development. Emerging evidence suggests that β-CAs, in addition to being involved in photosynthesis, also contributed to plant defense against pathogens (12). Fungal β-CAs are essential for growth, differentiation, survival, and virulence (13). Research has found that in hemiascomycetous yeasts, β-CA is required for fungal growth specifically under CO_2_ conditions (14, 15). Among many pathogenic fungi in humans, such as *Candida albicans*, *Cryptococcus neoformans*, *Cryptococcus gattii*, *Aspergillus fumigatus,* and *Aspergillus nidulans*, β-CAs are critical for pathogenicity (14, 16–18).

Opportunistic and pathogenic fungi can sense the environmental CO_2_ levels, which influence their virulence or environmental subsistence traits. CAs are essential for fungal growth because they provide bicarbonate for HCO_3_^−^-dependent metabolic carboxylation reactions, such as those catalyzed by acetyl-CoA carboxylase, carbamoyl-phosphate, and pyruvate carboxylase synthetase (19). The fungal CO_2_-sensing is directly stimulated by HCO_3_^−^ produced in a CA-dependent manner, which directly activates adenylyl cyclase (AC) involved in fungal spore formation (20). In the fungal pathogens *C. albicans* and *C. neoformans*, AC was shown to transmit the CO_2_ signal in a CA-dependent manner (14, 16, 21). The catalytic domains of *C. albicans* and *C. neoformans* ACs were demonstrated to be activated by HCO_3_^−^ *in vitro* (14, 21). CAs may be considered as novel “pathogen proteins” that represent potential targets for antifungal therapies (20). Moreover, CAs are required to establish sexual reproduction in basidiomycetes and filamentous ascomycetes and that various isoenzymes of filamentous ascomycetes have been discovered in the cytoplasm and mitochondria (19).

Mitochondria serve as the primary site of cellular respiration, where organic molecules are oxidized via the tricarboxylic acid (TCA) cycle and accompanying reactions. The oxidative phosphorylation (OXPHOS) system facilitates the transfer of electrons from NADH or FADH2 to molecular oxygen (22). CAs can have a central role in adjusting the proton concentration in the mitochondrial matrix, ensuring the efficient operation of ATP synthase for ATP production (23). In protozoans, algae, and plants, the mitochondrial NADH-dehydrogenase complex of the respiratory chain (complex I) includes a CA module attached to its membrane arm on the matrix side. Recent research shows that the CA module may directly provide protons (H^+^) for translocation across the inner mitochondrial membrane at complex I. Researchers suggest that CAs anchoring in complex I represent the original configuration to secure OXPHOS in the context of early endosymbiosis (23). The mammalian carbonic anhydrases VA (CA VA) and VB (CA VB) are the only two enzymes that are expressed in the mitochondria. The main function of CA VA is to provide HCO_3_^−^ ions to the enzymes that are involved in several important biochemical pathways in mitochondria, which play an important role in ammonia detoxification (ureagenesis) through carbamoyl phosphate synthetases I (24).

Nitrogen is one of the most essential nutrients for biological activities. The regulation of nitrogen metabolism is a complex and delicate process. In most legume nodules, the carbonic anhydrase (CA)-phosphoenolpyruvate carboxylase (PEPC)-malate dehydrogenase (MDH) pathway constitutes a key route for nitrogen assimilation (25). In the actinomycete-plant symbiotic system, CA-mediated cytoplasmic acidification helps maintain ammonium retention, thereby promoting nitrogen fixation (26). In *Mesorhizobium loti*, CA also contributes to the formation of a buffering environment in the periplasm, facilitating the protonation of NH₃ and optimizing its transmembrane transport and assimilation (27). Fungi are frequently exposed to nitrogen-limited environments, making effective regulation of nitrogen metabolism crucial for their survival, growth, development, and pathogenicity (28). In addition, CA also participates in biosynthetic or detoxification pathways where HCO_3_⁻ serves as a cofactor or substrate, such as the degradation of cyanate, which provides an additional nitrogen source for fungi (29, 30). Amino acids, as organic compounds with significant biological functions, play a crucial role in various processes such as protein synthesis, cell growth, development, and energy production and are closely related to nitrogen metabolism. Multiple sources of glutamate (Glu) biosynthesis contribute to maintaining glutamate homeostasis. Glutamate homeostasis not only plays a critical role in central nitrogen metabolism but also coordinates various key metabolic functions (31).

*Magnaporthe oryzae* is the causal agent of rice blast disease, which causes huge yield losses in rice production globally each year (32). Numerous studies have demonstrated that nutrient acquisition during infection and the synthesis of primary metabolite components, such as amino acids, are crucial for full pathogenicity in *M. oryzae* (33–36). In *M. oryzae*, the glutamine synthetase MoGln1 is essential for autophagy, fungal virulence, and development (31). However, at present, the research on the relationship between CAs and nitrogen metabolism in *M. oryzae* is still relatively limited, and there are few related reports. Our previous research has indicated that the β-carbonic anhydrase (MoCA1) of *M. oryzae* is involved in the pathogenic mechanism of the blast fungus (37). In this study, we verified the interactions among MoCA1 and other carbonic anhydrases (MoCAs) within the mitochondria of *M. oryzae*. Our findings reveal that the mitochondrial carbonic anhydrase family in *M. oryzae* not only participates in the growth of conidia and fungal pathogenicity but also contributes to mitochondrial function and ATP synthesis. Furthermore, these enzymes are involved in the regulation of glutamine (Gln)-glutamate (Glu) metabolic pathways and the expression of nitrogen metabolism-related genes. Our results highlight the crucial role of the carbonic anhydrase family in maintaining intracellular pH homeostasis, providing significant insights into how this pathogen adapts to the hostile environments within the host during infection.

## MATERIALS AND METHODS

### Fungal strains, culture conditions, and plasmids

*M. oryzae* strain JJ88 was used as wild-type (WT) and was isolated and purified from *Oryza sativa* cultivar Jijing88, a variety that is widely planted in Jilin Province, China. All fungal strains were cultured on CM agar plates and stored on filter paper at –20°C. For the stress of carbonic anhydrase inhibitors, the strain was inoculated on potato dextrose agar (PDA) medium and cultured in the dark at 28°C for 7 days. For conidia, the strain was inoculated onto oatmeal agar medium (OMA) and incubated for 7 days in the dark at 28°C. After washing with sterile distilled water to remove the aerial hyphae of the strain, the strain was cultured in the dark for another 3 days. The vector construction was carried out using Luria Bertani (LB) medium. Ingredients of media used are as follows: potato dextrose agar medium (12.0 g·L⁻¹ potato extract powder, 20.0 g·L⁻¹ glucose, 14.0 g·L⁻¹ agar, pH 5.6 ± 0.2), complete medium ( 10 g·L⁻¹ glucose, 1 g·L⁻¹ Ca(NO_3_)_2_·4H_2_O, 1 g·L⁻¹ yeast extract, 0.5 g·L⁻¹ enzymatic hydrolyzed casein, 0.5 g·L⁻¹ acid hydrolyzed casein, 0.2 g·L⁻¹ KH_2_PO_4_, 0.25 g·L⁻¹ MgSO_4_·7H_2_O, 0.15 g·L⁻¹ NaCl, and 16.0–18.0 g·L⁻¹ agar), oatmeal agar medium ( 30.0 g·L⁻¹ oatmeal, 15.0–20.0 g·L⁻¹ agar), Luria Bertani medium (10.0 g·L⁻¹ tryptone, 5.0 g·L⁻¹ yeast extract, 10.0 g·L⁻¹ sodium chloride, pH 7.0 ± 0.1), and minimal medium (MM, 10 g·L⁻¹ glucose, 2 g·L⁻¹ K_2_HPO_4_, 1.5 g·L⁻¹ KH_2_PO_4_, 0.5 g·L⁻¹ MgSO_4_·7H_2_O, 0.1 g·L⁻¹ CaCl_2_·6H_2_O, 0.0025 g·L⁻¹ FeSO_4_ ·7H_2_O, 0.5 g·L⁻¹ (NH_4_)_2_SO_4_, 0.15 g·L⁻¹ NaCl, and 16.0–18.0 g·L⁻¹ agar).

*Escherichia coli* DH5α (Sagon, Shanghai) was used for all vector plasmid construction. The vectors pGADT7 and pGBKT7 were used in yeast two-hybrid assays, with *Saccharomyces cerevisiae* AH109 (Coolaber, China) as the host strain. The plasmid pKD7-Red, encoding a red fluorescent protein (RFP), was used for subcellular localization experiments. YN/YC vectors (pEarleyGate201/202) were employed for Gateway seamless cloning. The AGL-1 strain was used for transformation of *M. oryzae. Agrobacterium tumefaciens* strain GV3101 (Weidi Bio-Technology Co., Shanghai) was used for transformation of *Nicotiana benthamiana*. All plasmids were maintained in our laboratory.

### Assays for the subcellular localization of MoCA2, MoCA4*,* and MoCA6

The *BamH* I-*BamH* I, *Sma* I-*Sma* I, and *BamH* I-*Sma* I sites of the vector pKD7-Red containing RFP were used to tag the localization of MoCA2, MoCA4, and MoCA6, respectively. Subsequently, we generated transgenic strains expressing RFP-labeled fusion genes of *MoCA2*, *MoCA4*, and *MoCA6* in the deletion mutants of *M. oryzae* (*pKD7-MoCA2:: RFP*, *pKD7-MoCA4:: RFP*, and *pKD7-MoCA6::RFP*). Hyphae (6 days old) and conidia (3 days old) from transformants carrying the fusion gene with RFP were observed under a fluorescence microscope. To observe the mitochondria in hyphae and conidia, 1 mM Mito-Tracker Green (Yuanye Bio-Technology Co., Shanghai) solution was used and incubated at 37°C for 20–45 min, followed by observation under a laser scanning confocal microscope (Olympus fluoview FV3000, Japan).

### Yeast two-hybrid (Y2H) assay

Using restriction endonuclease to cleave the full-length coding sequences of MoCA1, MoCA2, MoCA4, and MoCA6, PCR amplification was performed, and sequencing verification was performed. The obtained DNA fragments of MoCA2, MoCA4, and MoCA6 were cloned into the pGADT7 vector to form a fusion plasmid, which was verified by PCR. To clarify the interaction between MoCA1 and MoCA2, MoCA1 and MoCA4, MoCA1 and MoCA6 proteins, MoCA1-pGBKT7 was used as bait and further transformed into fusion strains with MoCA2-pGADT7, MoCA4-pGADT7, and MoCA6-pGADT7 fusion plasmids in yeast strain AH109. The target bands were verified by PCR, and Y2H yeast cells carrying these three pairs of fusion strains were grown on SD/- Trp/- Leu and SD/- Trp/- Leu/- His with X-α-gal plates.

### Bimolecular fluorescence complementation (BiFC) assay

The coding sequences of MoCA1 and MoCA2, MoCA4, and MoCA6 were introduced into the vectors pearlygate201 (YN) and pearlygate202 (YC) via LR recombination reactions to generate recombinant plasmids MoCA1-YN, MoCA2-YC, MoCA4-YC, and MoCA6-YC, respectively. These recombinant plasmids were transformed into *A. tumefaciens* GV3101 and plated on LB medium containing Kana and Rif, followed by incubation at 28°C for 2–3 days. Positive clones were verified by PCR and coinjected into 4-week-old *N. benthamiana* leaves at 1:1. The leaves were kept in the dark for 12–16 h and then exposed to light for 2–3 days. The leaf samples were then incubated with JC-1 mitochondrial dye at 37°C for 20–30 min and observed for yellow fluorescent protein (YFP) fluorescence signals under a confocal microscope (Olympus fluoview FV3000, Japan).

### Targeted gene deletion and complementation

To create knockout constructs for *MoCA2*, *MoCA4*, and *MoCA6*, the upstream and downstream fragments of each gene were amplified using specific primer pairs. The PCR products were then cloned into the pXEH2.0 vector to generate the constructs pXEH2.0-MoCA2, pXEH2.0-MoCA4, and pXEH2.0-MoCA6. These constructs were introduced into the AGL-1 strain of *A. tumefaciens* and transformed into the WT *M. oryzae* using the *Agrobacterium*-mediated transformation method as previously described (37). Transformants were selected on the medium containing 200 μg/mL hygromycin and identified by PCR using specific primers.

The entire *MoCAs* (*MoCA2*, *MoCA4*, and *MoCA6*) sequences were amplified using a PCR technique with *MoCAs*_gene-F/R and inserted into the hygromycin-resistant vector pKD7 for complementation into the mutant strain, respectively. The reconstructed pKD7-MoCAs were transformed into the Δ*MoCAs* (Δ*MoCA2*, Δ*MoCA4*, and Δ*MoCA6*) mutant strains and designated Δ*MoCAs*/*CAs* (Δ*MoCA2*/*CA2*, Δ*MoCA4*/*CA4*, and Δ*MoCA6*/*CA6*). The complemented strain was confirmed by PCR with MoCAs_gene-F/R.

To further verify the gene deletion and complementation, the expression of the WT, Δ*MoCAs* mutants, and Δ*MoCAs/CAs* strains was amplified using quantitative real-time PCR (qRT-PCR) with MoCAs_qF/R and Actin_F/R, and the strains were identified. The primers for gene deletion and complementation are listed in Table S1.

### Determination of the growth of knockout mutant strains under CA inhibitor conditions

Wild-type and *MoCA1*, *MoCA2*, *MoCA4*, *MoCA6* knockout mutant strains were cultured in the PDA medium containing CA inhibitor-acetazolamide (Ace) (MCE, China) (38) at a concentration of 50 nM in the experimental group and 0 nM in the control group at a temperature of 28°C, and the lesion area was counted on the 7th day.

### Determination of the growth of knockout mutant strains under nitrogen starvation and supplementation conditions

WT and knockout mutant strains cultured in abundant-nitrogen medium (MM) and nitrogen-free medium (MM-N) to explore the growth status of the strains in the absence of nitrogen sources. Glutamine and glutamic acid as nitrogen sources were allocated at a concentration of 100 mM and then filtered and sterilized, and the amino acid solution was added to the MM-N medium configured in advance at a concentration of 90% at a ratio of 10% so that amino acids were used as the only nitrogen source in the medium and the final concentration was 0.2 mM. The strains were inoculated in the above medium, inverted in an incubator at 28°C for 7 days.

### qRT-PCR

Total RNA was extracted from 7-day-old fungal colonies grown on PDA medium using the Spin Column Fungal Total RNA Purification Kit (Sangon Biotech). For cDNA synthesis, All-in-One 5X RT MasterMix (Applied Biological Materials Inc.) was used to reverse transcribe 2 μg of total RNA from each strain. qRT-PCR was subsequently performed using the CFX96 Optical Reaction Module (BIO-RAD, Singapore) and BlasTaqTM 2×qPCR MasterMix (Applied Biological Materials Inc.). The relative mRNA levels were calculated using the 2^−ΔΔCq^ (Cq=Cq_gene_−Cq_actin_) method (39). The *M. oryzae* actin gene (*MGG_03982.6*) was used as the internal control gene for normalization. Each experiment included three technical replicates for each sample. The primer sequences used for qRT-PCR are shown in Table S1.

### Assays for conidial production, growth, and development

WT, knockout mutant strains, and complementary strains were cultured on OMA media as previously described (37). Following 3 days of cultivation at 28°C, sterile water was added to remove the hyphae. A piece of the culture medium was cut with a blade and placed on a glass slide, which was then incubated at 28°C in a humidifying chamber. The prepared samples were examined under a Nexcope microscope (NE620) at 12, 24, 48, and 72 h. Thereafter, the strains were stained with lactophenol cotton blue to observe the conidiophore stalks and hyphae under a Nexcope microscope 35. Moreover, conidia were collected with 2 mL of sterile water after 3 days of culture on OMA media and counted using a hemocytometer. Each strain was repeated three times, and the experiment was performed in triplicate.

Conidia of the WT, knockout mutant strains, and complementary strains were cultured on OMA media to observe the germination of conidia and the formation of appressoria. The conidial suspensions were adjusted to 1 × 10^–^⁵/mL and added dropwise to a hydrophobic cover slip under a microscope at 1, 2, 3, 4, and 6 h. For turgor pressure, 2–4 M glycerol was used to treat the appressoria for 3 min at 14 and 24 h post-inoculation (hpi), and at least 100 appressoria were counted to observe their collapse rate (40). Three biological replicates of each strain were used, and the experiment was carried out in triplicate.

### Rice sheath penetration and plant infection assays

To assess the virulence of the knockout strains, the WT, knockout mutant strains, and complementary strains were cultured on OMA media to harvest the conidia as previously detailed. The fourth leaf stage of rice seedlings (*O. sativa* cv. Lijiangxintuanheigu was tested for infection after being sprayed with 2 mL of a conidial suspension (5 × 10⁴ conidia/mL in 0.2% gelatin). The inoculated plants were kept in the dark at 28°C for 24 h and subsequently moved to a growth chamber with a 16-h light cycle for 7 days following inoculation (dpi). The severity of lesions was analyzed by quantification of the *M. oryzae* genomic 28S rDNA relative to rice genomic Rubq1 DNA (41). Conidial suspensions (100 μL, 5 × 10⁴ conidia/mL) were introduced into seedling leaf sheaths and maintained in a humidified chamber as previously outlined. At 24, 48, and 72 hpi, the average growth rates of infectious hyphae (IH) and their spread to neighboring cells were examined under a Nexcope microscope (NE620) for 100 germinated conidia per treatment (42). This procedure was conducted three times. The experiment was carried out with three independent replicates, and the representative outcomes from one of these trials are shown.

### Determination of the ATP content of knockout strains

The production of ATP was monitored using an ATP content detection (Nanjing Jiancheng Bioengineering Institute) kit to determine the diversity of ATP content in the mycelium of *M. oryzae*.

### JC-1 mitochondrial membrane potential assay

To verify the mitochondrial membrane potential damage in the WT strain and the knockout mutant strains, conidia of the WT, Δ*MoCA2*, Δ*MoCA4*, and Δ*MoCA6* strains that had been cultured on OMA medium for 5–7 days were collected using the aforementioned method. The mitochondrial membrane potential was then detected using the JC-1 mitochondrial membrane potential detection kit (LABLEAD, China) (38) and observed under a confocal microscope (Olympus fluoview FV3000, Japan).

## RESULTS

### MoCAs are mitochondrial carbonic anhydrases that interact with MoCA1

**Fig 1:**
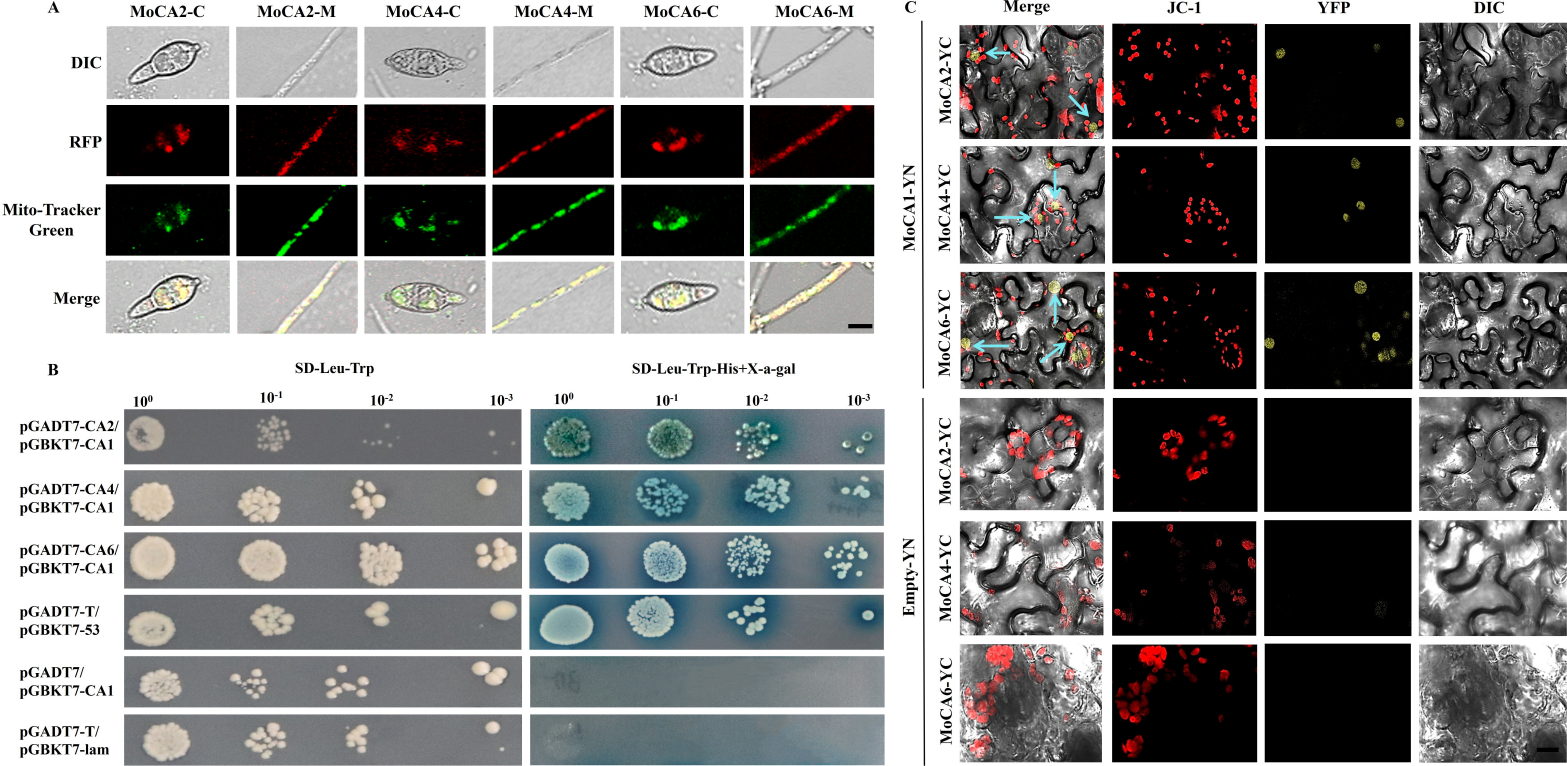
MoCA2, MoCA4, and MoCA6 subcellular localization, interaction with MoCA1, and function in *Magnaporthe oryzae*. (**A**) Subcellular localization in hyphae and conidia. Red fluorescence signals of MoCA2-RFP, MoCA4-RFP, and MoCA6-RFP protein of hyphae and conidia were examined by confocal microscopy and colocalized with Mito-Tracker Green on mitochondria. Scale bar = 10 μm. (**B**) Y2H between MoCA2, MoCA4, MoCA6, and MoCA1. MoCA2-AD, MoCA4-AD, MoCA6-AD, and MoCA1-BD were co-transformed into yeast strain Y2H Gold and cultured on SD−Leu−Trp and SD−Leu−Trp−His media. pGADT7-MoCA2/pGBKT7-MoCA1, pGADT7-MoCA4/pGBKT7-MoCA1, and pGADT7-MoCA6/pGBKT7-MoCA1, experimental group; *pGADT7-T/pGBKT7-53*, positive control group; pGADT7/pGBKT7-MoCA1, negative control group; *pGADT7-T/pGBKT7-lam*, blank group. (**C**) BiFC assay demonstrating the interaction between MoCA2, MoCA4, MoCA6, and MoCA1. YFP fluorescence signals were observed in *N. benthamiana* leaves following co-expression of MoCA2-YC, MoCA4-YC, and MoCA6-YC (C-terminal fragment of YFP) with MoCA1-YN (N-terminal fragment of YFP). JC-1 staining confirmed mitochondrial membrane localization. Negative controls included MoCA2-YC, MoCA4-YC, MoCA6-YC, and YN alone. Scale bar = 5 μm.

In the preliminary study, we used the UniProt website (https://www.uniprot.org/uniprotkb) and the SMART website (https://smart.embl.de/smart/change_mode.cgi) to predict and analyze the related genes. We found that the three genes, *MGG_04973*, *MGG_06600*, and *MGG_09234*, encode zinc-coordinated CAs, each containing a CA domain at amino acid positions 39–263, 25–184, and 26–169, respectively (Fig. S1A through C). These genes were named *MoCA2*, *MoCA4*, and *MoCA6*, respectively (three genes abbreviated as *MoCAs*). To investigate the protein interactions of these three genes, we generated subcellular localization transformants of MoCA2, MoCA4, and MoCA6 fused with RFP (Fig. S2). By comparing with Mito-Tracker Green-labeled mitochondria, we observed that MoCA2, MoCA4, and MoCA6 were localized in the mitochondria of the hyphae and conidia (Fig. 1A). Similarly, the zinc-coordinated carbonic anhydrase MoCA1 is also localized in the mitochondria (37). Therefore, we hypothesized that MoCA1 may interact with MoCA2, MoCA4, and MoCA6.

To verify the interaction between MoCA1 and these three proteins, we employed Y2H technology (Fig. S3) and BiFC technology (Fig. S4). The results showed that in the Y2H experiment, only the experimental group and the positive control transformed with the target plasmid formed colonies and turned blue on SD Trp/Leu/His medium containing X-α-gal, while the blank control and negative control remained colorless (Fig. 1B), confirming the interaction between MoCA1 and MoCA2, MoCA4, and MoCA6.

To further clarify whether MoCAs interact with MoCA1 within the mitochondria, the BIFC assay demonstrated that when MoCA1-YN was co-transformed with MoCAs-YC in tobacco, the interaction between MoCA1 and MoCAs resulted in YFP, as indicated by the mitochondrial dye JC-1 (red fluorescence), showing their interaction within the mitochondria (Fig. 1C). In contrast, the empty vector YN did not produce YFP when co-transfected with MoCAs in the mitochondria (Fig. 1C). These results confirm that MoCA1 interacts with MoCA2, MoCA4, and MoCA6 within the mitochondria of the cell.

### MoCAs exhibit functional synergy

**Fig 2:**
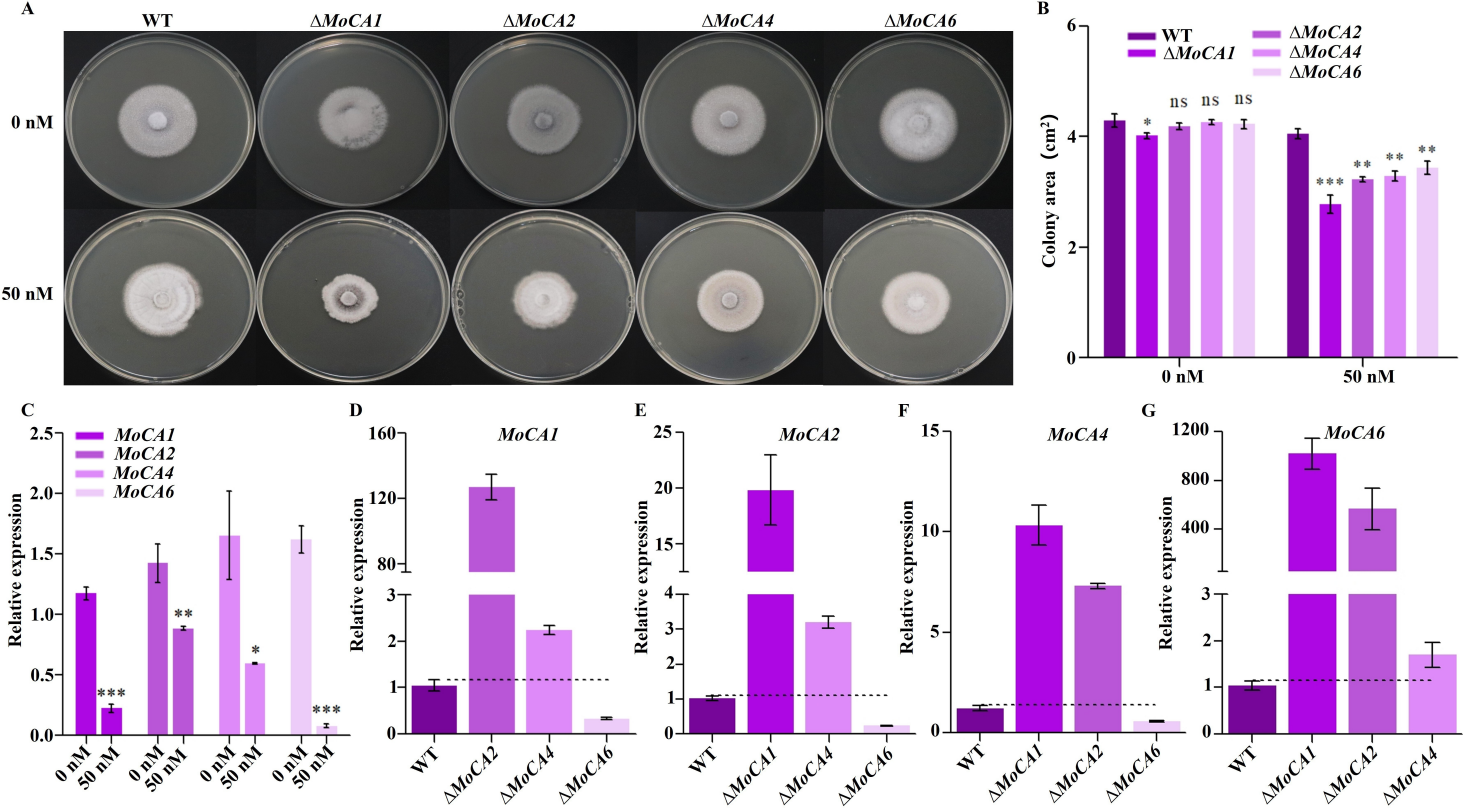
Analysis of synergistic effects of *MoCA* genes. (**A**) Colony morphology of WT, ∆*MoCA1*, and ∆*MoCA2*, ∆*MoCA4*, and ∆*MoCA6* on media with 0 nM and 50 nM carbonic anhydrase inhibitor. (**B**) Colony area measurement and statistical analysis. (**C**) Statistical analysis of *MoCA* gene expression in ACE of the WT strain. (**D**) Statistical analysis of *MoCA1* gene expression in WT, Δ*MoCA2*, Δ*MoCA4*, and Δ*MoCA6*. (**E**) Statistical analysis of *MoCA2* gene expression in WT, Δ*MoCA1*, Δ*MoCA4*, and Δ*MoCA6*. (**F**) Statistical analysis of *MoCA4* gene expression in WT, Δ*MoCA1*, Δ*MoCA2*, and Δ*MoCA6*. (**G**) Statistical analysis of *MoCA6* gene expression in WT, Δ*MoCA1*, Δ*MoCA2*, and Δ*MoCA4*. Error bars represent mean ± SD from three replicates. ns, *P* > 0.05; *P < 0.05, **P < 0.01, and ***P < 0.001.

To further investigate the functional synergy among *MoCA2*, *MoCA4*, and *MoCA6* genes, we generated knockout mutants Δ*MoCA2*, Δ*MoCA4*, and Δ*MoCA6* in the WT rice blast fungus (Fig. S5). Ace is a small heterocyclic sulfonamide that binds with high affinity to various CAs and acts as a CA inhibitor (38). We cultured the WT, Δ*MoCA1*, Δ*MoCA2*, Δ*MoCA4*, and Δ*MoCA6* strains on PDA medium supplemented with 0 nM and 50 nM Ace. At 0 nM, there was no significant difference in growth between the WT and the knockout strains, except for Δ*MoCA1*. However, at 50 nM, although the growth of the WT strain was largely unaffected, the growth of the Δ*MoCA1*, Δ*MoCA2*, Δ*MoCA4*, and Δ*MoCA6* knockout strains was significantly inhibited (Fig. 2A and B). Since *MoCA1* has been confirmed to have CA activity (37), the phenotypic changes observed in the knockout strains under 50 nM Ace further validate the prediction that *MoCA2*, *MoCA4*, and *MoCA6* belong to the carbonic anhydrase family. Additionally, we compared the expression levels of *MoCA1*, *MoCA2*, *MoCA4*, and *MoCA6* in the WT strain under both culture conditions. We found that, at 50 nM Ace, the expression levels of *MoCA1*, *MoCA2*, *MoCA4*, and *MoCA6* in the WT were significantly lower than those under 0 nM Ace (Fig. 2C), suggesting potential functional compensation among the *MoCAs*. To further validate this result, we examined the expression levels of other existing *MoCA* genes in the WT, Δ*MoCA1*, Δ*MoCA2*, Δ*MoCA4*, and Δ*MoCA6* strains (Fig. 2D through G). Surprisingly, we observed increased expression of these genes in the mutants, particularly the notable upregulation of *MoCA6* in other mutant backgrounds (Fig. 2G), further demonstrating the functional synergy among the *MoCAs*.

### MoCAs are involved in conidial development in *M. oryzae*

**Fig 3:**
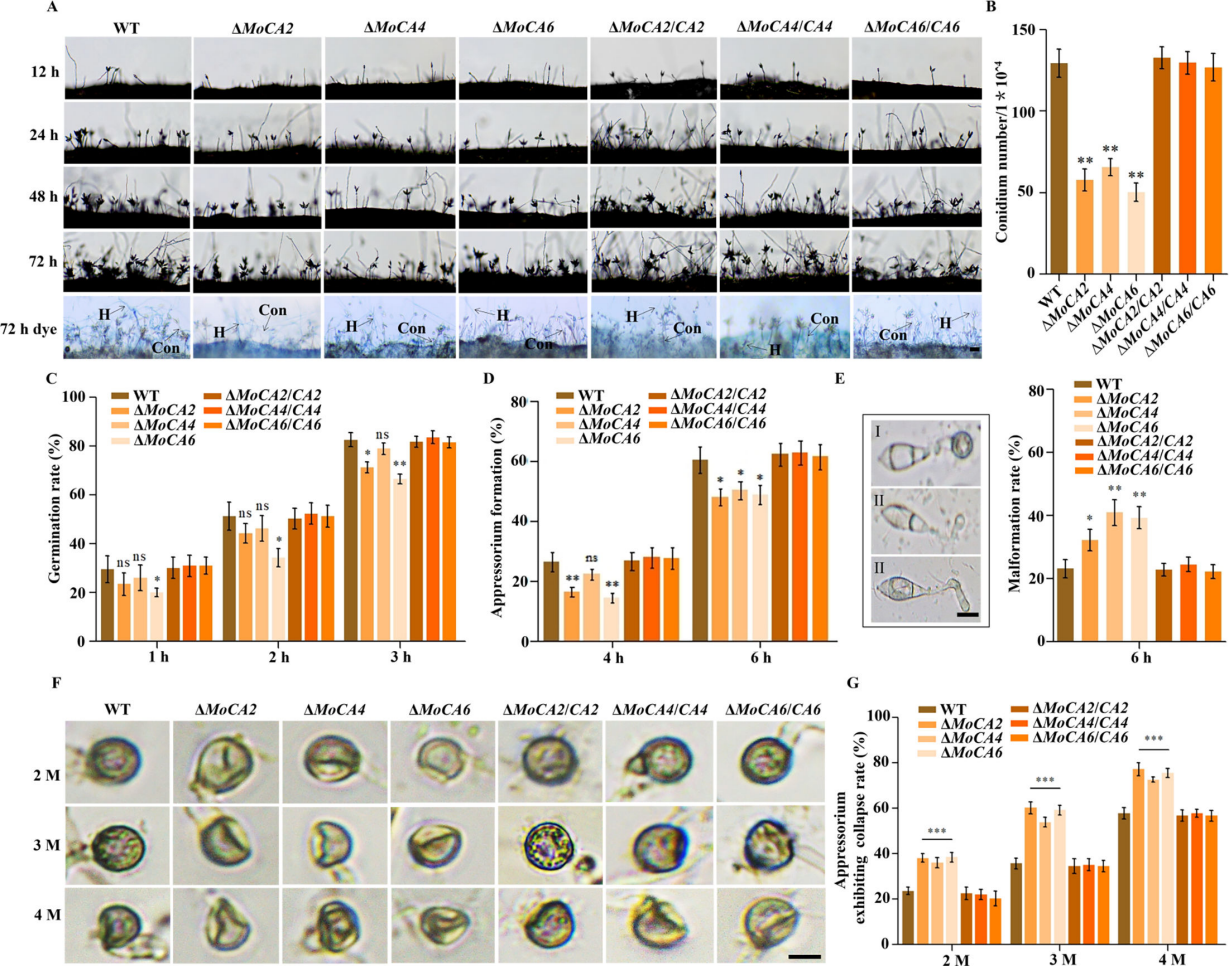
Conidia and appressoria development analysis of the WT, mutant strains, and complementary strains. (**A**) Observation time points for conidiophores of WT, Δ*MoCAs*, and Δ*MoCAs*/*CAs* strains were 12, 24, 48, and 72 h. Conidiophores were stained with lactophenol cotton blue at 72 h. Hyphae (H) were stained blue, and conidiophore (Con) stalks gray. Scale bar = 50 μm. (**B**) Statistical analysis of conidia numbers among WT, Δ*MoCAs*, and Δ*MoCAs*/*CAs* strains. (**C**) Conidial germination rate. Conidial germination was calculated under the microscope at 1, 2, and 3 h. (**D**) Appressorial formation rate. Appressorial formation was calculated under the microscope at 4 and 6 h. (**E**) The statistics of the appressorial malformation rate. I, normal appressoria; II, malformed appressoria. Scale bar = 10 μm. (**F**) Observation for turgor pressure of the WT, Δ*MoCAs*, and Δ*MoCAs*/*CAs* strains was done in 2–4 M glycerol. Scale bar = 10 μm. (**G**) Appressorium collapse rate. Error bars represent mean ± SD from three replicates. ns, *P* > 0.05; *P < 0.05, **P < 0.01, and ***P < 0.001.

To investigate the biological phenotypes of the *MoCAs* in *M. oryzae*, we conducted studies on conidial development. The results showed that the number of conidiophores and conidia was significantly reduced in the Δ*MoCA2*, Δ*MoCA4*, and Δ*MoCA6* mutants compared to the WT and the restored transformants throughout the developmental process, as evidenced by staining analysis (Fig. 3A). When the conidial counts were quantified at 3 dpi, the numbers of conidia produced by Δ*MoCA2*, Δ*MoCA4*, and Δ*MoCA6* were significantly lower than half of the WT and the restored transformants (Fig. 3B).

In addition, we compared the germination and appressorium formation of conidia to the Δ*MoCAs* mutants, the WT, and the restored transformants (Fig. S6A). The results showed that at 1–3 h, the germination rate of Δ*MoCA4* conidia was slightly lower than that of the WT and the restored transformants, but the difference was not statistically significant. However, the germination rates of Δ*MoCA2* and Δ*MoCA6* conidia were significantly lower than that of the WT and the restored transformants at 3 h (Fig. 3C). Similarly, during appressorium formation, the formation rate of Δ*MoCA4* was slightly lower than that of the WT and the restored transformants at 4 h, but the difference was not significant. In contrast, the formation rates of Δ*MoCA2* and Δ*MoCA6* were significantly lower than that of the WT and the restored transformants, reaching approximately two-thirds of the WT and the restored transformant level (Fig. 3D). At 6 h, the appressorium formation rates of all three mutants were significantly lower than that of the WT and the restored transformants (Fig. 3D). Furthermore, we analyzed the incidence of abnormal appressoria at 6 h. As shown in Fig. 3E, normal appressoria were classified as type I, while abnormal appressoria were classified as type II. Compared to the WT and the restored transformants, the incidence of abnormal appressoria was significantly increased in the knockout mutants, exceeding 35% (Fig. 3E). The above results are consistent with the expression profiles at different developmental stages (Fig. S6B through D). Turgor pressure experiments revealed that the appressorium collapse rate of the knockout mutants was significantly higher by 25%–30% in 2–4 M glycerol compared to the WT and the complemented strains (Fig. 3F and G).

These results indicate that the *MoCA2*, *MoCA4*, and *MoCA6* genes are involved in the formation of conidiophores, conidia, and appressoria in *M. oryzae*.

### MoCAs are important for pathogenicity in *M. oryzae*

**Fig 4:**
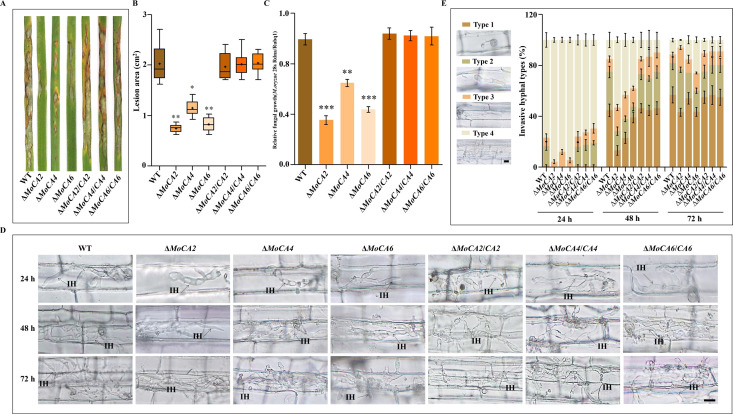
Pathogenicity and invasive hyphal growth mechanisms of *MoCA2*, *MoCA4*, and *MoCA6*. (**A**) Spray inoculation assay on rice leaves. Conidia suspensions of the WT, Δ*MoCAs*, and Δ*MoCAs*/*CAs* were used to infect rice leaves. (**B**) Lesion area analysis. (**C**) Statistical analysis of relative fungal growth. (**D**) Rice leaf sheath infection assay. The conidial suspension of indicated strains was injected into a rice sheath. Representative photographs of infectious hyphae were taken after 24, 48, and 72 h of incubation at 25°C. Scale bar = 10 μm. IH, infectious hyphae. (**E**) The infection rate was calculated according to the number of type 1 to type 4 events. The infection status of more than 100 germinated conidia per leaf sheath was scored at 24, 48, and 72 hpi. Type 1 has no penetration; type 2 only has a penetration peg or a single infectious hypha (IH); type 3 has more than two IH in one rice cell; type 4 has extensive IH into adjacent rice cells. Error bars represent at least three independently repeated standard deviations. Error bars represent at least three independently repeated standard deviations. ns, *P* > 0.05, *P < 0.05, **P < 0.01, and ***P < 0.001.

To investigate the impact of appressorium maturation and morphological defects on the pathogenicity of *MoCAs* mutants, we conducted pathogenicity assays using conidia collected from WT, Δ*MoCAs,* and Δ*MoCAs*/*CAs* strains on rice leaves and sheaths. After inoculating healthy rice seedlings with conidial suspensions, significant disease lesions were observed on rice leaves inoculated with WT, Δ*MoCAs,* and Δ*MoCAs*/*CAs* conidia at 7 dpi. Compared to the WT and the complementary strains, the lesion areas caused by Δ*MoCA2*, Δ*MoCA4*, and Δ*MoCA6* conidia were significantly reduced (Fig. 4A). Statistical analysis of the lesion area and relative fungal growth revealed that the pathogenicity of Δ*MoCA2*, Δ*MoCA4*, and Δ*MoCA6* strains was significantly lower than those of the WT and the complementary strains (Fig. 4B and C).

In addition, sheath infection assays were performed to evaluate the invasive expansion ability of WT, Δ*MoCAs,* and Δ*MoCAs*/*CAs* strains within rice cells. Microscopic observation showed that the invasive capacity of Δ*MoCA2*, Δ*MoCA4*, and Δ*MoCA6* conidia in sheath cells was significantly weaker compared to that of the WT and Δ*MoCAs*/*CAs* strains (Fig. 4D). To further clarify the specific roles of *MoCA2*, *MoCA4*, and *MoCA6* in pathogenic development, we defined four types of infectious hyphae based on their developmental morphology (Fig. 4E). Subsequently, we quantified the proportions of the four types of infectious hyphae among 100 infected conidia in the sheath (Fig. 4E). At 24 hpi, approximately 28% of WT conidia developed into invasive and primary infectious hyphae, whereas less than 15% of Δ*MoCA2*, Δ*MoCA4*, and Δ*MoCA6* conidia formed primary infectious hyphae. At 48 hpi, the number of type 2 and 3 invasive hyphae in Δ*MoCA2*, Δ*MoCA4*, and Δ*MoCA6* strains was significantly lower than that in the WT and Δ*MoCAs*/*CAs* strains. Furthermore, at 72 hpi, although the invasive hyphae of Δ*MoCA2*, Δ*MoCA4*, and Δ*MoCA6* extended toward neighboring cells, the infection level of Δ*MoCA2* and Δ*MoCA6* remained significantly lower than that of the WT and Δ*MoCAs*/*CAs* strains (Fig. 4E).

These results demonstrate that *MoCA2*, *MoCA4*, and *MoCA6* are essential for the pathogenicity of *M. oryzae*, and their deletion significantly impairs the invasive capacity and pathogenicity of the fungal strains.

### MoCAs are involved in ATP synthesis in mitochondria

**Fig 5:**
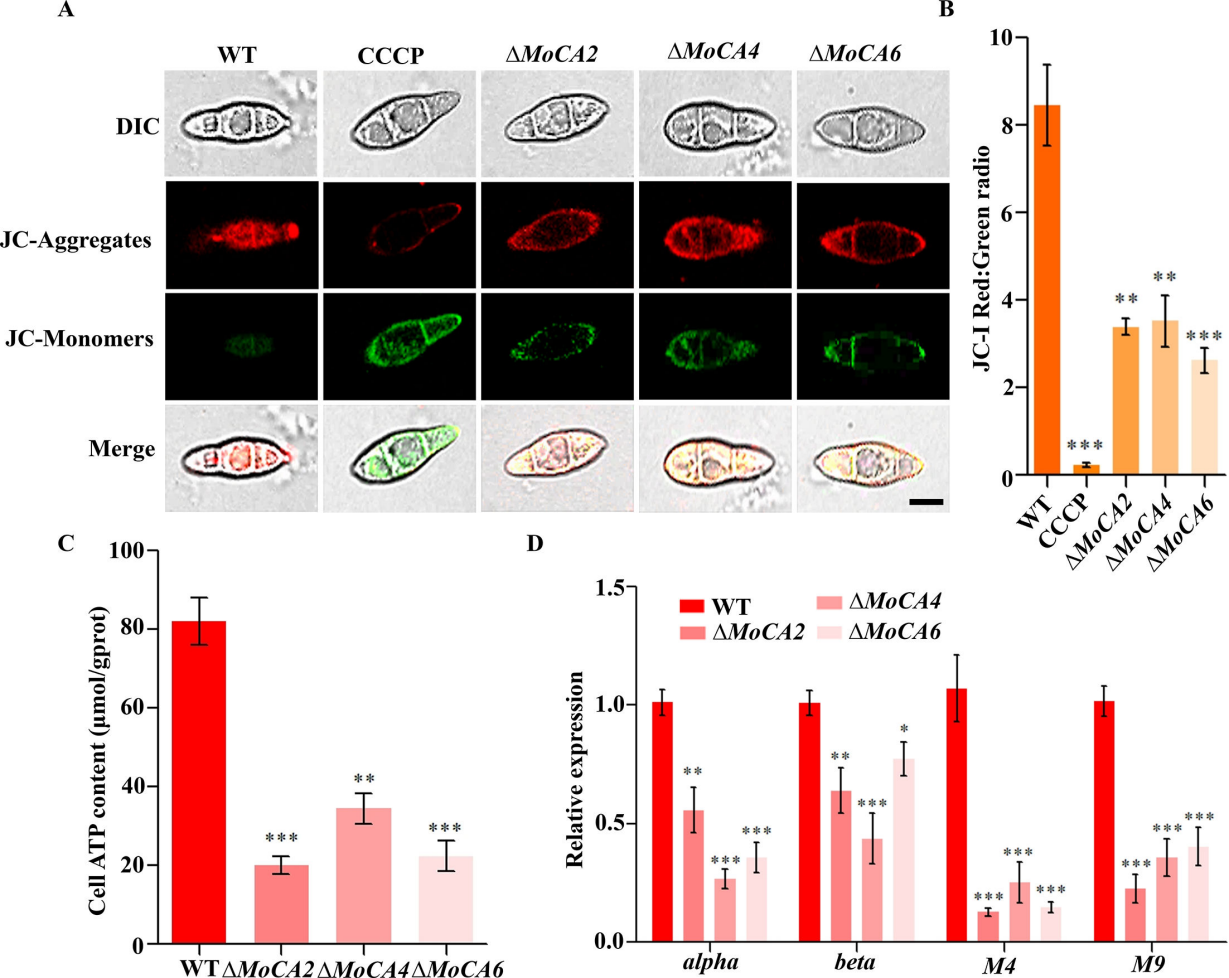
The role of *MoCA2*, *MoCA4,* and *MoCA6* in ATP synthesis. (**A**) Mitochondrial membrane potential test using the JC-1 dye. Confocal microscopy was used for observation, with CCCP as a positive control. Scale bar = 10 µm. (B）Ratio of red to green fluorescence intensity indicating mitochondrial membrane potential. (**C**) Statistical analysis of the ATP content of mutant strains. (**D**) Statistical analysis of ATP synthase-related gene expression in WT and knockout strains of *M. oryzae*. Error bars represent mean ± SD from three replicates. *P < 0.05, **P < 0.01, and ***P < 0.001.

Mitochondria are essential organelles for maintaining normal cellular physiological functions. A decrease in mitochondrial membrane potential (MMP) is a hallmark of early-stage apoptosis and an important indicator for evaluating mitochondrial function (43). Previous studies have shown that MoCAs are localized in mitochondria (Fig. 1A). To further clarify the roles of MoCAs in mitochondria, we used the JC-1 probe to assess changes in mitochondrial membrane potential in different strains. JC-1 forms J-aggregates in the mitochondrial matrix and emits red fluorescence, while green fluorescence is weak or almost undetectable. As shown in Fig. 5A, the conidia of the WT strain exhibited normal mitochondrial membrane potential (Δψm) and red fluorescence. In contrast, the Δ*MoCA2*, Δ*MoCA4*, and Δ*MoCA6* knockout strains, as well as the CCCP positive control group, showed significantly increased green fluorescence (indicating mitochondrial dysfunction), with the CCCP group showing more pronounced effects. Using ImageJ software, we analyzed the red-to-green fluorescence ratio of JC-1 and found that the ratio in Δ*MoCA2*, Δ*MoCA4*, and Δ*MoCA6* strains was significantly lower than that in the WT strain, even less than half of the WT value (Fig. 5B). These results indicate that the gene deletion of *MoCA2*, *MoCA4*, and *MoCA6* significantly reduced the mitochondrial Δψm in *M. oryzae*.

Mitochondria are the primary site for ATP synthesis. ATP synthase subunit 4 (M4) and ATP synthase subunit 9 (M9) are important components of the ATP synthase in mitochondria, while ATP synthase subunits α (α) and β (β) are essential parts of the ATP synthase complex (44). Subsequently, we analyzed the ATP content and the expression levels of the α, β, M4, and M9 ATP synthase genes in the WT and Δ*MoCAs* strains. The results showed that the ATP content in Δ*MoCA2*, Δ*MoCA4*, and Δ*MoCA6* strains was significantly reduced compared to that in the WT strain (Fig. 5C). Furthermore, the expression levels of all four ATP synthase genes were markedly lower in the knockout mutants than in the WT strain (Fig. 5D).

In summary, the deletion of *MoCA2*, *MoCA4*, and *MoCA6* leads to a decline in mitochondrial function in *M. oryzae*, suggesting that these three genes may play a synergistic role in ATP synthesis.

### MoCAs participate in the Gln-Glu nitrogen metabolism pathway in *M. oryzae*

Nitrogen is an essential nutrient for fungal growth. The ability of fungi to metabolize various nitrogen sources enables them to colonize different ecological niches and survive under nutrient-limited conditions (45). In the preliminary study, we predicted that the genes *MoCA4* and *MoCA6* may be involved in the nitrogen metabolism pathway using the KEGG database (https://www.genome.jp/kegg/kegg2.html). Further analysis revealed that the genes *MGG_02593*, *MGG_06062*, *MGG_06888*, and *MGG_08074* are associated with the metabolism of nitroalkane (involved in nitrite-forming), nitrate (involved in the reduction of nitrate to nitrite), glutamine synthetase (involved in the synthesis of glutamine) (46), and glutamate dehydrogenase (involved in the synthesis of glutamate), respectively (Fig. S7).

**Fig 6:**
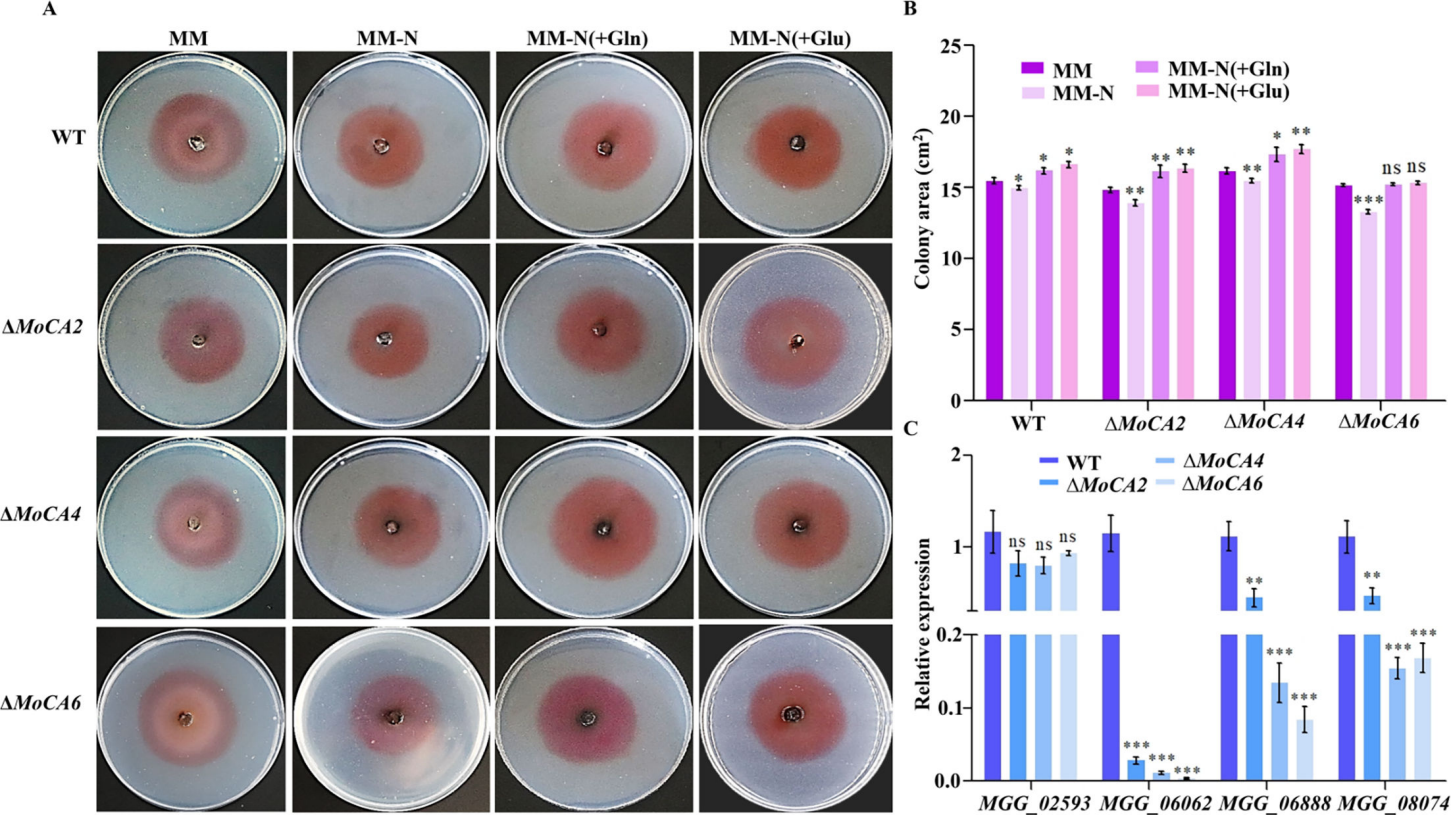
The role of *MoCA2, MoCA4,* and *MoCA6* in nitrogen metabolism. (**A**) Strains inoculated in MM (abundant-nitrogen medium), MM-N (nitrogen-free medium), MM-N (+Gln) (nitrogen-free medium with glutamic acid), and MM-N (+Glu) (nitrogen-free medium with glutamine). The hyphae were stained with Congo red dye and photographed for images. (**B**) Statistical analysis of colony size after 7 days of cultivation in abundant-nitrogen medium, nitrogen-free medium, nitrogen-free medium with glutamic acid, and nitrogen-free medium with glutamine. (**C**) Statistical analysis of gene expression in nitrogen metabolism of WT, Δ*MoCA2*, Δ*MoCA4*, and Δ*MoCA6*. Error bars represent at least three independently repeated standard deviations. ns, *P* > 0.05; *P < 0.05, **P < 0.01, and ***P < 0.001.

To further investigate the role of *MoCA2*, *MoCA4*, and *MoCA6* in nitrogen metabolism, WT and corresponding knockout mutants were inoculated onto media with MM, MM-N, MM-N (+Gln), and MM-N (+Glu), respectively. The results showed that in the nitrogen-deficient medium (MM-N), the growth of all strains was significantly inhibited compared to that in the MM medium. However, the growth restriction in the mutant strains was more pronounced than in the WT strain. When glutamine (Gln) or glutamate (Glu) was added to the nitrogen-deficient medium, the growth capacity of the strains was restored, and in some strains, it even exceeded that observed in the MM (Fig. 6A and B).

Subsequently, the expression levels of four key nitrogen metabolism-related genes (*MGG_02593*, *MGG_06062*, *MGG_06888*, and *MGG_08074*) were analyzed. The results showed no significant difference in the expression of *MGG_02593* between WT and the knockout mutants. However, the expression levels of *MGG_06062*, *MGG_06888*, and *MGG_08074* were significantly lower in the knockout mutants compared to those in the WT (Fig. 6C).

These findings suggest that *MoCA2*, *MoCA4*, and *MoCA6* not only function independently in the glutamine-glutamate nitrogen metabolism pathway but also interact with other genes to collectively regulate the entire nitrogen metabolic network.

## DISCUSSION

Carbonic anhydrases (CAs) are widely distributed enzymes that catalyze the reversible hydration of carbon dioxide to form bicarbonate and protons (10). Due to the presence of metal ions in the active site, these enzymes are classified as metalloenzymes. Zn²^+^ is one of the most widely used metal cofactors in nature. It does not participate in redox reactions but acts as a Lewis acid by accepting a pair of electrons. The widespread occurrence of Zn²^+^ in all CA families further highlights its unique properties (47). Studies have shown that MoCAs are all Zn²^+^-dependent carbonic anhydrases. Among them, MoCA2 belongs to the α-carbonic anhydrase family, while MoCA4 and MoCA6 belong to the β-carbonic anhydrase family. In α-carbonic anhydrases, the metal ion in the active site is typically coordinated by three histidine (His) residues, whereas in β-carbonic anhydrases, it is coordinated by one His residue and two cysteine (Cys) residues (48). Notably, MoCA1 is a β-carbonic anhydrase that is coordinated by three highly conserved amino acid residues: Cys46, His102, and Cys105 (37). Sequence prediction and alignment analysis revealed that the three highly conserved amino acid residues in MoCA4 and MoCA6 are Cys37, His93, Cys97 and Cys38, His91, Cys94, respectively (Fig. S1D). In contrast, MoCA2, an α-CA, was aligned with α-carbonic anhydrases from *Dunaliella salina* and *Chlamydomonas reinhardtii* (10), and its three highly conserved residues were identified as His126, His128, and His145 (Fig. S1E). Some transition metals can replace Zn²^+^ in the active site or bind to His and Cys residues outside the active site, thereby inhibiting CA activity. Previous studies have investigated this phenomenon in various tissues of different animal species (47). Therefore, future research could focus on developing antimicrobial agents targeting the inhibition of Zn²^+^ or the active site of MoCAs by such metals. As shown in Fig. 2, *MoCAs* exhibit functional interactions. Interestingly, the relative expression levels of the other genes in the Δ*MoCA6* mutant were significantly lower than those observed in the WT, while the expression of *MoCA6* was markedly upregulated in all the mutant strains (Fig. 2G), suggesting that *MoCA6* may play a central role within the carbonic anhydrase family. Further investigations into the protein-protein interactions among other carbonic anhydrase genes are warranted to better understand the potential synergistic effects within this gene family.

α- and β-type carbonic anhydrases play essential physiological roles in protozoa, fungi, and bacteria, ensuring the availability of bicarbonate for metabolism or participating in pH regulation (10). CAs are critical for the survival, proliferation, and virulence of bacteria, enabling them to thrive in dynamically changing organic nutrient environments (49). Similarly, CAs play important roles in the CO₂ sensing systems and the regulation of sexual development in fungal pathogens. In *Sordaria macrospora*, the mitochondrial-localized carbonic anhydrase CAS2 is essential for providing HCO_3_⁻ during early development, which is crucial for regulating the activity of adenylyl cyclase SAC1, thereby participating in the regulation of nutritional growth, ascospore germination, and fruiting body formation (19). Based on these findings, we have demonstrated that *MoCA* genes localize to the mitochondria in both hyphae and conidia (Fig. 1A). Nevertheless, we will further track their subcellular localization in invasive hyphae to gain insights into the spatial distribution of these proteins during host infection. And we further validated that the *MoCA* genes are involved in conidia growth and development (Fig. 2), regulating key genes related to conidia growth and development, thus participating in the normal development process of *M. oryzae* conidia.

CAs have emerged as a key factor in the virulence of various microorganisms. One of the critical mechanisms by which CAs enhance virulence is through the regulation of intracellular pH. Many pathogenic microorganisms require specific acidic or pH conditions to infect host tissues (50). By maintaining an optimal pH environment, CA facilitates the survival and proliferation of pathogens within host cells, thereby sustaining the infection process (51). CAs are involved in multiple aspects of host-pathogen interactions, including bacterial adhesion to host tissues, invasion of host cells, and modulation of immune responses (52, 53). In pathogenic fungi such as *C. albicans* and *C. neoformans*, CAs play a crucial role in development and virulence (54). In these two fungal species, AC relies on CAs to transmit CO₂ signals, participating in CO₂ sensing and virulence regulation (14, 16). Combined with the findings of this study, the deletion of *MoCA* genes significantly impaired the infection ability and pathogenicity of the fungal strain (Fig. 4), suggesting that *MoCAs* may function synergistically in the pathogenesis of *M. oryzae*. We hypothesize that *MoCAs* may regulate intracellular pH homeostasis, thereby activating AC and modulating the cAMP signaling pathway to influence the pathogenicity of *M. oryzae*. To further validate this hypothesis, it is recommended to use experimental approaches to monitor the dynamic changes in intracellular pH during the infection of WT and knockout mutants in the host, in order to clarify how *MoCAs* regulate intracellular pH homeostasis during the pathogenic process.

Mitochondria are the primary organelles responsible for cellular respiration in aerobic living cells and are often referred to as the “powerhouse” of the cell. In addition to this core function, mitochondria are also involved in various physiological processes, including phospholipid biosynthesis, amino acid metabolism, cell death, signal transduction, metabolic homeostasis, and innate immunity (23, 55). Mitochondrial dysfunction, typically characterized by a loss of membrane potential, leads to insufficient energy supply and increased oxidative stress (56, 57). Studies have shown that the mitochondrial membrane potential is significantly reduced in Δ*MoCAs* knockout strains (Fig. 5A and B), indicating that the *MoCA* gene family plays a crucial role in maintaining mitochondrial function.

Mitochondria serve as a critical site for ATP synthesis. The proton motive force generated by the electrochemical gradient ultimately drives ATP synthase, facilitating the conversion of adenosine diphosphate (ADP) and inorganic phosphate into ATP through the process of OXPHOS. This process not only ensures ATP production but also maintains the cellular redox state, particularly the reoxidation of NADH (22, 58). The NADH dehydrogenase complex (Complex I) plays a key role in the mitochondrial respiratory chain. It serves as the primary entry point for electrons in the mitochondrial electron transport chain (mETC), and thus is essential for ATP generation. In *Arabidopsis thaliana*, carbonic anhydrasea CA1 and CA2 are components of the plant-specific “carbonic anhydrase domain” within Complex I of the mitochondrial respiratory chain, and they play a regulatory role in ATP production (59). Moreover, the intracellular ATP content and the expression levels of ATP synthesis-related genes are markedly decreased in these knockout mutants (Fig. 5C and D), further supporting this conclusion. Previous studies have revealed a pathogenic model mediated by the MoAE4/MoCA1 system (37, 60). Among them, MoCA1 exerts its catalytic activity within mitochondria, while the HCO_3_⁻ transporter *MoAE4* is localized to the plasma membrane and vacuolar membrane, contributing to the maintenance of intracellular CO₂–HCO_3_⁻ homeostasis. Based on the results of this experiment, we further speculate that MoCA1 may form an interaction pattern with MoCA2, MoCA4, and MoCA6 in mitochondria and jointly participate in functional processes. Glucose molecules are metabolized in the mitochondria through the TCA cycle, generating CO_2_, H_2_O, and NADH as by-products. Under normal conditions, CO₂ and H₂O are catalyzed by the carbonic anhydrase family to undergo hydration reactions, producing H^+^ and HCO_3_⁻. Meanwhile, NADH participates in the OXPHOS process in the mitochondrial inner membrane. During this process, the carbonic anhydrase family collaborates to provide protons, thereby driving ADP to be converted into ATP under the action of ATP synthase. In this process, the carbonic anhydrase family not only supplies the necessary protons for ATP synthesis but also regulates the corresponding ATP synthase. Therefore, the carbonic anhydrase family collaborates in the maintenance of the intracellular CO_2_-HCO_3_⁻ system in mitochondria, ensuring cellular acid-base balance and providing protons for the mitochondrial respiratory chain, thus influencing ATP production.

Fungi are capable of responding to quantitative and qualitative changes in nitrogen availability through complex regulatory mechanisms, including nitrogen sensors, signaling cascades (such as the TOR cascade), transcription factors, and other potentially interacting regulatory proteins. In *Saccharomyces cerevisiae*, *Aspergillus nidulans*, and *Neurospora crassa*, these regulatory mechanisms affect gene expression, leading to physiological and morphological changes, as well as the activation of developmental processes (45). Unlike their plant hosts, fungi can preferentially utilize ammonium, glutamine, and glutamate as primary nitrogen sources, while also being able to assimilate nitrate, nitrite, other amino acids, and proteins as secondary nitrogen sources (28). The virulence of plant pathogenic fungi is regulated by multiple cellular pathways that respond to changes in the host environment. Nitrogen limitation has been identified as a key signal that triggers the expression of virulence genes within the host (61). This study demonstrates that *MoCAs* are involved in the glutamine-glutamate metabolism and regulate other nitrogen metabolism-related genes. Therefore, it is proposed that *MoCAs* may participate in the regulation of glutamine-glutamate metabolis*m in M. oryzae*, thereby maintaining intracellular nitrogen metabolic balance, ensuring the efficient utilization of nitrogen resources during infection.

Overall, this study provides experimental evidence for the role of the carbonic anhydrase family in mitochondria in coordinating intracellular pH homeostasis and glutamine-glutamate nitrogen metabolism, thereby influencing the growth, development, and pathogenicity of *M. oryzae*. It also offers new insights for the identification and development of novel targets for the control of rice blast.

### Conclusions

*M. oryzae* causes rice blast, one of the most devastating fungal diseases of rice, leading to significant global yield losses each year. To successfully invade the host and establish a stable parasitic relationship, *M. oryzae* must overcome various environmental stresses within the plant, including low nitrogen, high HCO_3_⁻, and hypoxic conditions. Therefore, maintaining a stable intracellular pH and metabolic environment is essential for its infection process. In this study, we found that the mitochondrial carbonic anhydrase family in *M. oryzae* not only participates in conidia growth and pathogenicity but also collaborates in the regulation of genes involved in ATP synthesis and glutamine-glutamate nitrogen metabolism. Our findings reveal the critical role of the carbonic anhydrase family in maintaining intracellular pH homeostasis, providing important insights into how pathogens adapt to the hostile environments within the host during infection.

## Data Availability

All gene sequences analyzed in this study were obtained from the *Magnaporthe orzyae* genome assembly available in the EnsemblFungi database (http://fungi.ensembl.org/Magnaporthe_oryzae/Info/Index).

## SUPPLEMENTAL MATERIAL

Supplemental material aem.02488-25-s0001.docx Table S1; Fig. S1 to S7.

ASM does not own the copyrights to Supplemental Material that may be linked to, or accessed through, an article. The authors have granted ASM a non-exclusive, world-wide license to publish the Supplemental Material files. Please contact the corresponding author directly for reuse.
