# Supplementary material for: Carbonic anhydrases contribute to mitochondrial function, conidial development, and pathogenicity of Magnaporthe oryzae
Source: Appl Environ Microbiol. 2026 Feb 12;92(3):e02488-25. doi: 10.1128/aem.02488-25 (PMC12997818; doi:10.1128/aem.02488-25)
Supplement: Supplemental material — Table S1; Fig. S1 to S7. [file aem.02488-25-s0001.docx]

**Carbonic anhydrases contribute to mitochondrial function, conidial development and pathogenicity of *Magnaporthe oryzae***

Qingqing Cui^1 *^, Tingzhen Wang^1 *^, Yujia Li^1^, Xiaojing Liang^1^, Xiaotong Song^1^, Xinyue Ji^1^, Linyi Wang^1^, Xinquan Wang^1^, Zhentao Chu^1^, Yuejia Dang^1 #^, Shi-hong Zhang^2 #^

^1^College of Life and Health, Institute of Modern Agriculture Research in Dalian University, Dalian 116000, China

^2^College of Plant Protection, Shenyang Agricultural University, Shenyang 110000, China

^#^Corresponding:

Yuejia Dang, E-mail: [dangyuejia@dlu.edu.cn;](mailto:dangyuejia@dlu.edu.cn;)

Shi-hong Zhang, E-mail: [zhangsh89@syau.edu.cn](mailto:zhangsh89@syau.edu.cn)

^*^These authors contributed equally to this work.

Qingqing Cui, E-mail: [cuiqingqing@dlu.edu.cn;](mailto:cuiqingqing@dlu.edu.cn;)

Tingzhen Wang, E-mail: [wangtingzhen@s.dlu.edu.cn](mailto:wangtingzhen@s.dlu.edu.cn).

**Declaration of competing interest**

The authors declare that the research was conducted in the absence of any commercial or financial relationships that could be construed as a potential conflict of interest.

**Declaration of generative AI and AI-assisted technologies in the writing process**

During the preparation of this work, the authors utilized ChatGPT-4 Turbo (developed by OpenAI) to assist with improving the readability and language of the manuscript. All content generated with the help of this tool was carefully reviewed and revised by the authors, who assume full responsibility for the final version of the published article.

**Supplementary Materials**

**Table S1. Primers used in this study**

| **Primer Name** | **Sequences (5’-3’)** | **Remarks** |
| --- | --- | --- |
| **MoCA2_upF** | CCGCTCGAGTATGGGTGCCTTGGCTTGT | Amplification of upstream fragment of *MoCA2* for the gene deletion |
| **MoCA2_upR** | GGAATTCACTGGGTGCGGGTTGCT |  |
| **MoCA2_downF** | ACGCGTCGACGGGCTGTGAGTTGGAT | Amplification of downstream fragment of *MoCA2* for the gene deletion |
| **MoCA2_downR** | CCCAAGCTTAGAAGGCAGGAGATGG |  |
| **MoCA4_upF** | GAAGATCTCCATCCATCGGGCACA | Amplification of upstream fragment of *MoCA4* for the gene deletion |
| **MoCA4_upR** | GGAATTCGGGTTATCGGCGGGTT |  |
| **MoCA4_downF** | CGGGATCCCCAGACCAGCAGAAGC | Amplification of downstream fragment of *MoCA4* for the gene deletion |
| **MoCA4_downR** | CCCAAGCTTCGATGCCACAAGTGAA |  |
| **MoCA6_upF** | GAAGATCTATGGGCTTACCGTTTC | Amplification of upstream fragment of *MoCA6* for the gene deletion |
| **MoCA6_upR** | GGAATTCGTAGGATTCCGCTTGG |  |
| **MoCA6_downF** | CGGGATCCGTGGGTGTTGGTGTTTATCG | Amplification of downstream fragment of *MoCA6* for the gene deletion |
| **MoCA6_downR** | CCCAAGCTTCGGTTGAACAAGGACGAGTA |  |
| **MoCA2_gene-F** | CGGGATCCATGAGAAATATCTTCAGCG | Using for PCR to confirm the deletion of *MoCA2* |
| **MoCA2_gene-R** | ACGCGTCGACTTTGAGGTTCTCGGCAG |  |
| **MoCA2_HYG-R-F** | GGTTGGCTTGTATGGAG |  |
| **MoCA2_HYG-R-R** | TGCCTGATTCGGATGG |  |
| **MoCA4_gene-F** | TCCCCCGGGATGGGCATGAGCGACG | Using for PCR to confirm the deletion of *MoCA4* |
| **MoCA4_gene-R** | TCCCCCGGGGCCGTTCAGCCAGTCC |  |
| **MoCA4_HYG-R-F** | TGGCAAACTGTGATGGA |  |
| **MoCA4_HYG-R-R** | GGCGGTAGTGTTAAGGA |  |
| **MoCA6_gene-F** | CGGGATCCATGGCAACTCCGAACCA | Using for PCR to confirm the deletion of *MoCA6* |
| **MoCA6_gene-R** | TCCCCCGGGGTCAACCCTCCTCGCCCTA |  |
| **MoCA6_HYG-R-F** | CTACCCAAGCATCCAAAT |  |
| **MoCA6_HYG-R-R** | GGACCCAGGACAAATCAG |  |
| **MoCA2_AD_F** | GGAATTCATGAGAAATATCTTCAGCG | Amplify *MoCA2* cDNA fragment for Y2H assay |
| **MoCA2_AD_R** | CGGGATCCTTTGAGGTTCTCGGCAG |  |
| **MoCA4_AD_F** | GGAATTCATGGGCATGAGCGACG | Amplify *MoCA4* cDNA fragment for Y2H assay |
| **MoCA4_AD_R** | TCCCCCGGGGCCGTTCAGCCAGTCC |  |
| **MoCA6_AD_F** | GGAATTCATGGCAACTCCGAACCA | Amplify *MoCA6* cDNA fragment for Y2H assay |
| **MoCA6_AD_R** | TCCCCCGGGGTCAACCCTCCTCGCCCTA |  |
| **MoCA1_BD_F** | CCGGAATTCATGGCTCAAAATCAGGATG | Amplify *Moca1* cDNA fragment for Y2H assay |
| **MoCA1_BD_R** | CGCGGATCCGCGAGCCGTCATAGAGGCG |  |
| **MoCA2_YC_F** | CGGGATCCATGAGAAATATCTTCAGCG | Amplification of the full length ORF of *MoCA2* for fusion with C-terminal YFP tag |
| **MoCA2_YC_R** | ACGCGTCGACTTTGAGGTTCTCGGCAG |  |
| **MoCA4_YC_F** | GCTCTAGA ATGAGAAATATCTTCAGCG | Amplification of the full length ORF of *MoCA4* for fusion with C-terminal YFP tag |
| **MoCA4_YC_R** | TCCCCCGGG GCCGTTCAGCCAGTCC |  |
| **MoCA6_YC_F** | CGGGATCCATGGCAACTCCGAACCA | Amplification of the full length ORF of *MoCA6* for fusion with C-terminal YFP tag |
| **MoCA6_YC_R** | TCCCCCGGGGTCAACCCTCCTCGCCCTA |  |
| **MoCA1_YN_F** | CTAGTCTAGAATGGCTCAAAATCAGGATG | Amplification of the full length ORF of *Moca1* for fusion with C-terminal YFP tag |
| **MoCA1_YN_R** | ACGCGTCGACGCGAGCCGTCATAGAG |  |
| **MGG_02593_qF** | GGGTCATCAGACACGC | qRT-PCR of *MGG_02593* |
| **MGG_02593_qR** | TCCGGTACCAGCAAAT |  |
| **MGG_06062_qF** | GATGGGCAAGTCTACG | qRT-PCR of *MGG_06062* |
| **MGG_06062_qR** | TTTCCGAGTGAATTGTC |  |
| **MGG_06888_qF** | GGCAGCATCGAGGAGTT | qRT-PCR of *MGG_06888* |
| **MGG_06888_qR** | GCCGGTAATCTGGTAAGG |  |
| **MGG_08074_qF** | GGCAGAACAGCTCGCG | qRT-PCR of *MGG_080745* |
| **MGG_08074_qR** | TGCTTCCGGCAACCAG |  |
| **Alpha_qF** | CGTGGTGAACGCTTGA | qRT-PCR for ATP synthase subunit alpha |
| **Alpha_qR** | TGAGGTGGGACAGGAA |  |
| **beta_qF** | TTTCCGATTTACTCAGGC | qRT-PCR for ATP synthase subunit beta |
| **beta_qR** | GGTGGTAATACGCTCTTG |  |
| **M4_qF** | TCATCAAGTTCGGTGGC | qRT-PCR for ATP synthase subunit 4 |
| **M4_qR** | TTGGAGACGGCAAAGAG |  |
| **M9_qF** | CGTGGTCAGCTTTTCA | qRT-PCR for ATP synthase subunit 9 |
| **M9_qR** | TGAGCAAGAAGGCAAC |  |
| **MoCA1_qF** | CTCAGATCCTCTGGCTTGGGT | qRT-PCR of *MoCA1* |
| **MoCA1_qR** | ACCCAAGCCAGAGGATCTGAG |  |
| **MoCA2_qF** | CGTCTTCCAAGCCGAAGACAAG | qRT-PCR of *MoCA2* |
| **MoCA2_qR** | GTGCTTAGACAGGCCACTGAAG |  |
| **MoCA4_qF** | GCCGTGGTTTAGCAAAATCC | qRT-PCR of *MoCA4* |
| **MoCA4_qR** | AGTCCTGCTGTAGACCAGAC |  |
| **MoCA6_qF** | GGTTTTAACCTGCATGGACG | qRT-PCR of *MoCA6* |
| **MoCA6_qR** | GTAAAGGTCAGCATGCCG |  |
| **Actin_qF** | CGTTGTTCCTATTTACGAGGG | qRT-PCR primer of *Actin* |
| **Actin_qR** | TTGATGTCACGGACGATTTC |  |
| **28S rDNA_qF** | TACGAGAGGAACCGCTCATTCAGATAATTA | qRT-PCR primer of 28S rDNA |
| **28S rDNA_qR** | TCAGCAGATCGTAACGATAAAGCTACTC |  |
| **Rubq1_qF** | GTGGTGGCCAGTAAGTCCTC | qRT-PCR primer of Rubq1 |
| **Rubq1_qR** | GGACACAATGATTAGGGATCA |  |


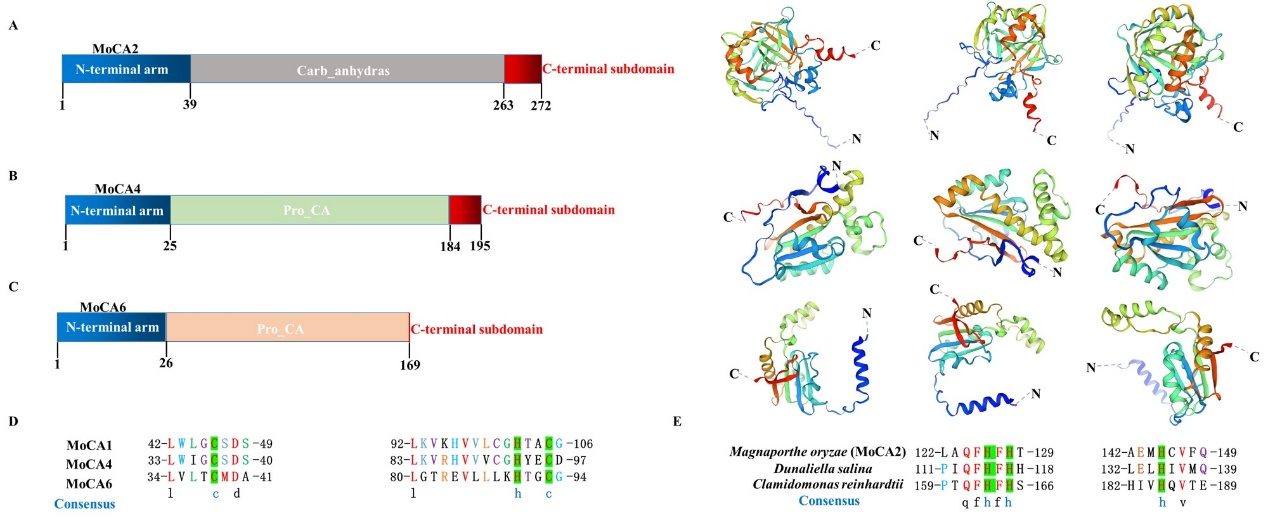


**FIG S1** Protein sequence analysis of MoCA2, MoCA4, and MoCA6. (A) Domain of MoCA2. MoCA2 is an α-carbonic anhydrase encoding zinc coordination and tertiary structure (3D) of MoCA2. (B) Domain of MoCA4. MoCA4 is an β-carbonic anhydrase encoding zinc coordination and tertiary structure (3D) of MoCA4. (C) Domain of MoCA6. MoCA6 is an β-carbonic anhydrase encoding zinc coordination and tertiary structure (3D) of MoCA6. N: N-Terminal C: C-Terminal. (D) Sequence alignment. A Zn ion coordinated by the three highly conserved residues two Cys and one His in MoCA1, MoCA4, and MoCA6. (E) Sequence alignment of α-CAs by the three highly conserved residues three His.


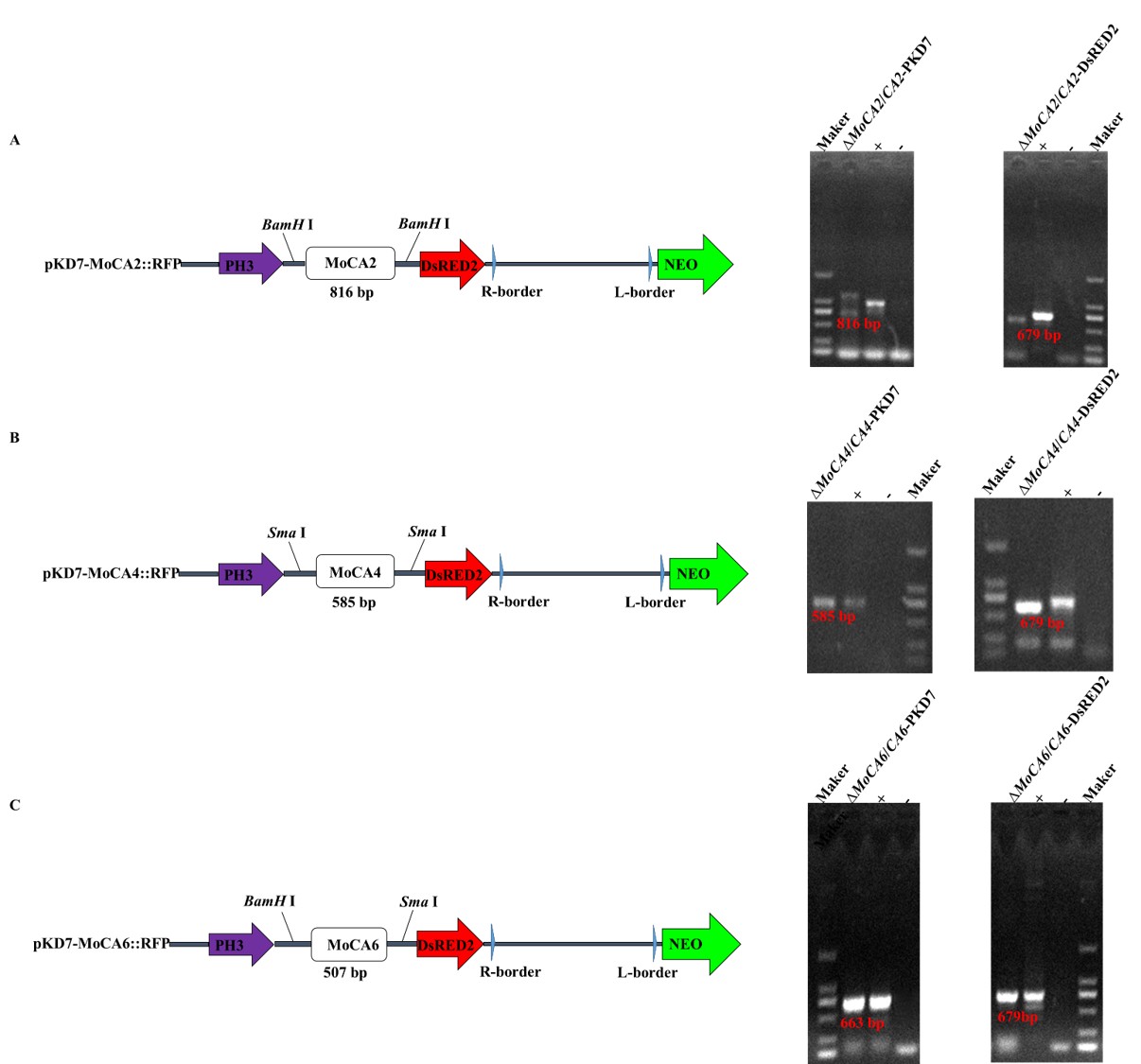


**FIG S2** Construction and validation of subcellular localization vectors for MoCA2, MoCA4, and MoCA6. (A) Construction of pKD7-MoCA2::RFP. Details of the construction are described in Materials and Methods. Validation of *MoCA2* complementary target gene bands. The target gene band was 816 bp. Δ*MoCA2*/*CA2* were Complementary mutants. +, wild type. -, ddH_2_O. (B) Construction of pKD7-MoCA4::RFP. Details of the construction are described in Materials and Methods. Validation of *MoCA4* complementary target gene bands. The target gene band was 585 bp. Δ*MoCA4*/*CA4* were Complementary mutants. +, wild type. -, ddH_2_O. (C) Construction of pKD7-MoCA6::RFP. Details of the construction are described in Materials and Methods. Validation of *MoCA6* complementary target gene bands. The target gene band was 507 bp. Δ*MoCA6*/*CA6* were Complementary mutants. +, wild type. -, ddH_2_O.


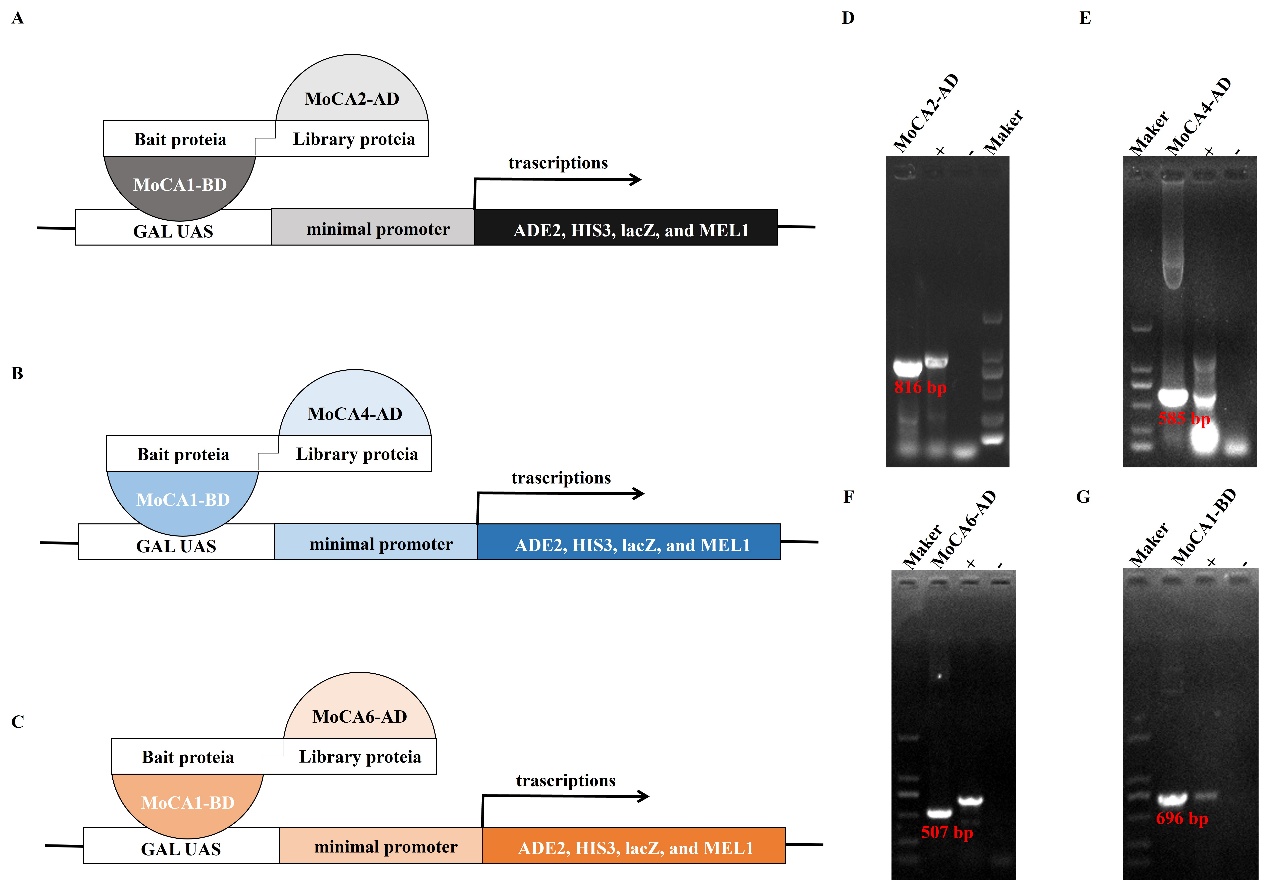


**FIG S3** Construction and validation of Y2H vectors for MoCA2, MoCA4, and MoCA6. (A) Construction diagram of Y2H yeast hybrid experimental vector for MoCA1 and MoCA2 protein interactions. (B) Verify the target gene band of MoCA2. The target gene band was 816 bp. +, wild type. -, ddH_2_O. (C) Construction diagram of Y2H yeast hybrid experimental vector for MoCA1 and MoCA4 protein interactions. (D) Verify the target gene band of MoCA4. The target gene band was 585 bp. +, wild type. -, ddH_2_O. (E) Construction diagram of Y2H yeast hybrid experimental vector for MoCA1 and MoCA6 protein interactions. (F) Verify the target gene band of MoCA6. The target gene band was 507 bp. +, wild type. -, ddH_2_O. (G) Verify the target gene band of MoCA1. The target gene band was 696 bp. +, wild type. -, ddH_2_O.


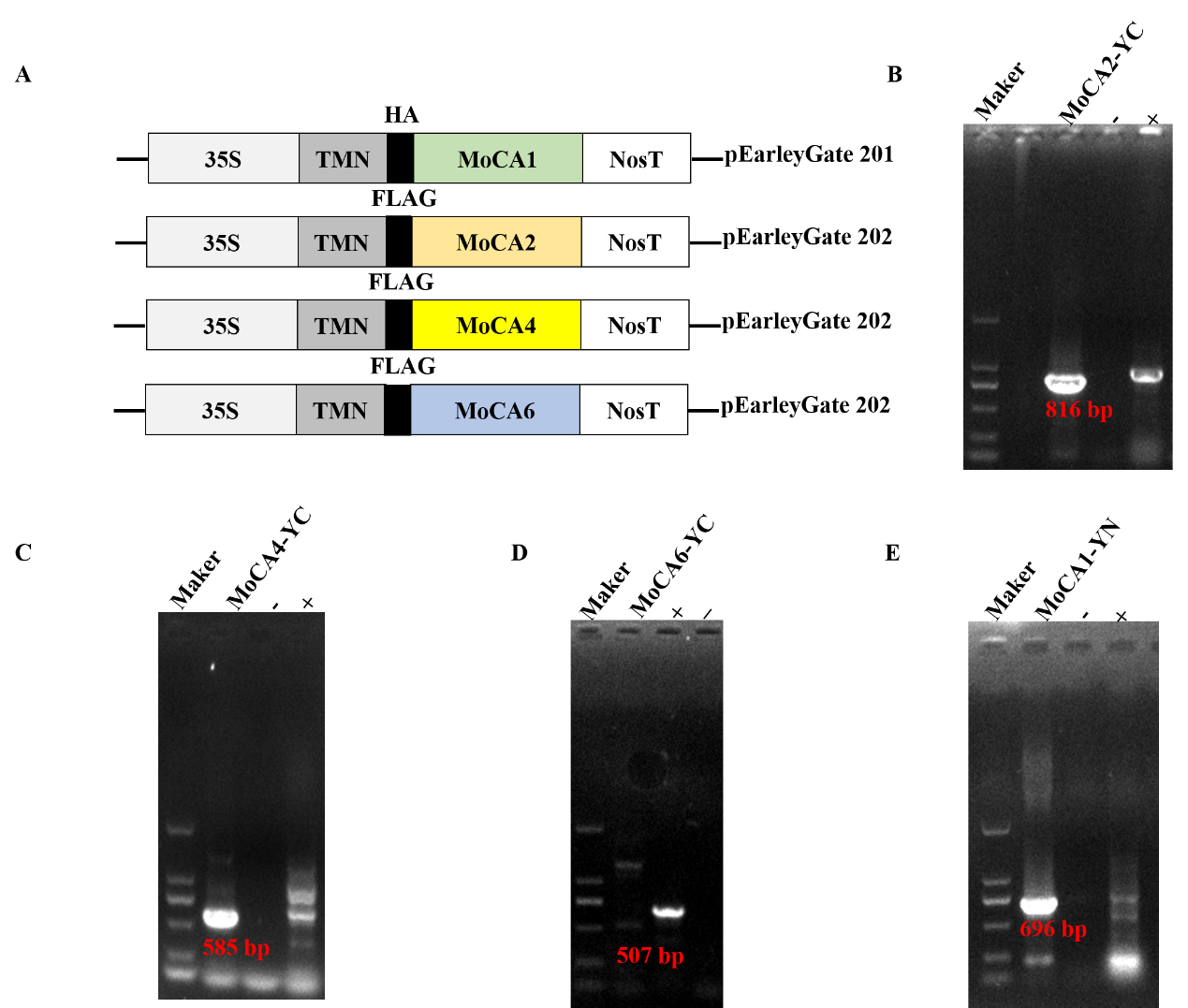


**FIG S4** Construction and validation of BiFC vectors for MoCA2, MoCA4, and MoCA6. (A) Construction diagram of a BiFC dual-fluorescent molecular experimental vector for MoCA1 and MoCA2, MoCA4, MoCA6 protein interactions. (B) Verification of the target gene band for MoCA2. The target gene band was 816 bp. +, wild type. -, ddH_2_O. (C) Verification of the target gene band for MoCA4. The target gene band was 585 bp. +, wild type. -, ddH_2_O. (D) Verification of the target gene band for MoCA6. The target gene band was 507 bp. +, wild type. -, ddH_2_O. (E) Verification of the target gene band for MoCA1. The target gene band was 696 bp. The target gene band was 696 bp. MoCA1 were protein. +, wild type. -, ddH_2_O.


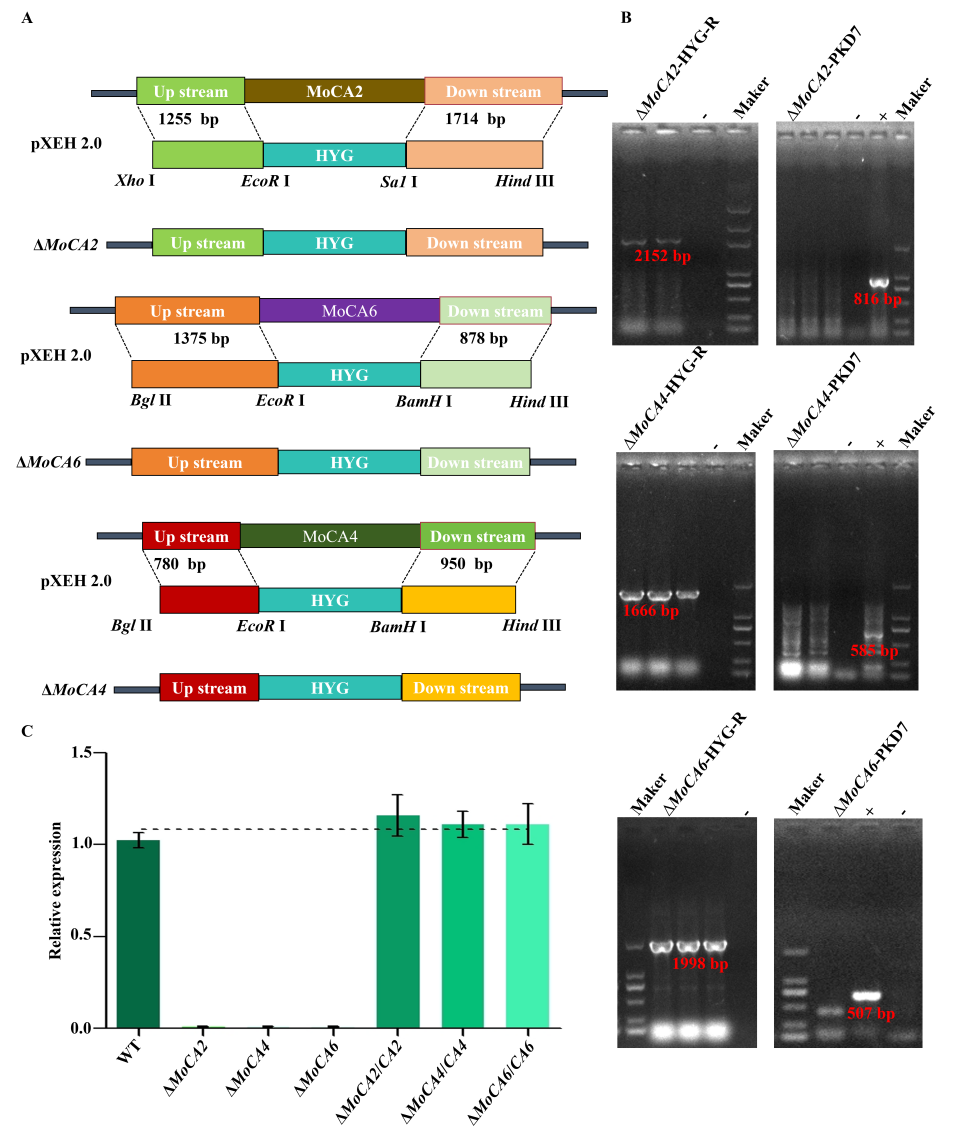


**FIG S5** Construction and validation of knockout vectors for the *MoCA2*, *MoCA4*, and *MoCA6* genes. (A) The *MoCAs* genes were replaced by the hph gene. (B) The flanking sequences were amplified with their corresponding primer pairs and fused with the HYG cassette. The target gene bands were detected. (C) Statistical analysis of *MoCAs* genes expression in WT, Δ*MoCAs*, and Δ*MoCA*/*CAs*.


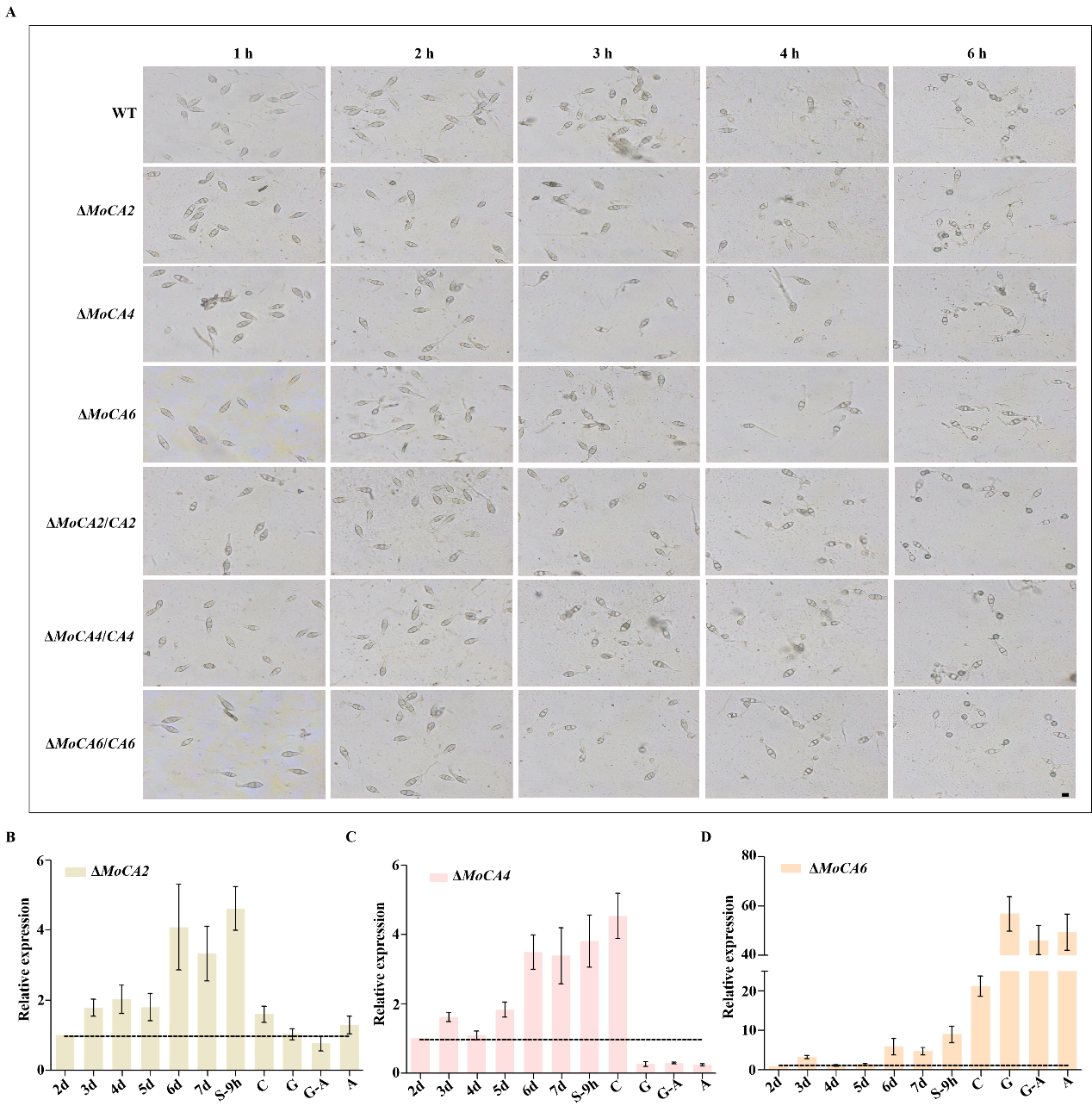


**FIG S6** Functional analysis and expression of the *MoCA2*, *MoCA4*, and *MoCA6* genes. (A) Comparison of conidia germination and adherent formation between Δ*MoCA2*, Δ*MoCA4*, and Δ*MoCA6* mutants and WT. Scale bar = 10 μm. (B) Expression of the *MoCA2* gene at different developmental stages in *M. oryzae*, using 2 d as a control. 2–7 d, hyphae; S-9 h, conidiophores; C, conidia; G, germination; G-A, germination and appressorium; A, appressorium. (C) Expression of the *MoCA4* gene at different developmental stages in *M. oryzae*, using 2 d as a control. 2–7 d, hyphae; S-9 h, conidiophores; C, conidia; G, germination; G-A, germination and appressorium; A, appressorium. (D) Expression of the *MoCA6* gene at different developmental stages in *M. oryzae*, using 2 d as a control. 2–7 d, hyphae; S-9 h, conidiophores; C, conidia; G, germination; G-A, germination and appressorium; A, appressorium. Error bars represent mean ± SD from three replicates.


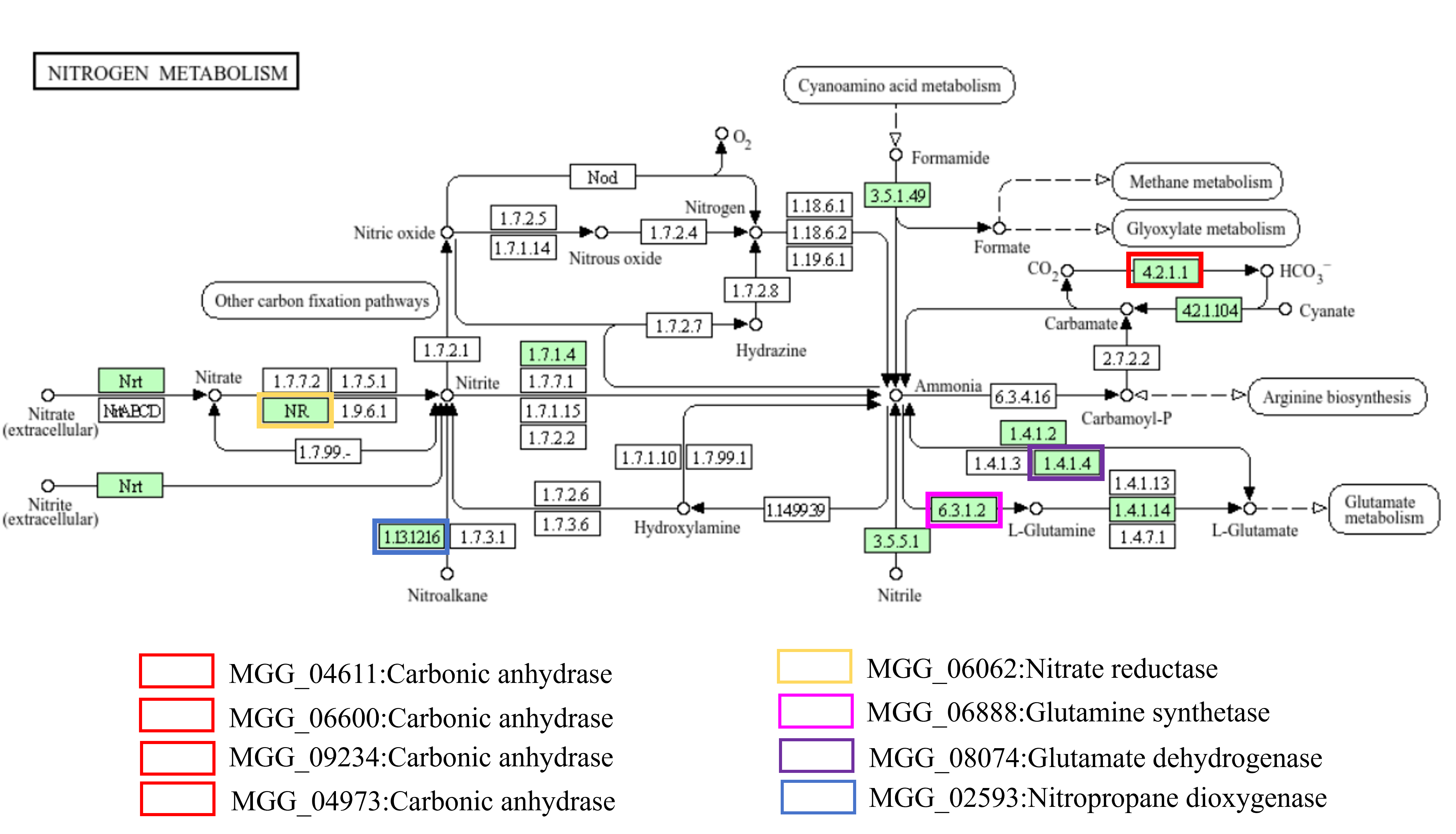


**FIG S7** Nitrogen metabolism pathway.
